# HIV-1 Reverse Transcriptase Promotes Tumor Growth and Metastasis Formation via ROS-Dependent Upregulation of Twist

**DOI:** 10.1155/2019/6016278

**Published:** 2019-12-02

**Authors:** Ekaterina Bayurova, Juris Jansons, Dace Skrastina, Olga Smirnova, Dzeina Mezale, Anastasia Kostyusheva, Dmitry Kostyushev, Stefan Petkov, Philip Podschwadt, Vladimir Valuev-Elliston, Sviataslau Sasinovich, Sergey Korolev, Per Warholm, Anastasia Latanova, Elizaveta Starodubova, Amir Tukhvatulin, Oleg Latyshev, Renat Selimov, Pavel Metalnikov, Alexander Komarov, Olga Ivanova, Tatiana Gorodnicheva, Sergey Kochetkov, Marina Gottikh, Ilze Strumfa, Alexander Ivanov, Ilya Gordeychuk, Maria Isaguliants

**Affiliations:** ^1^NF Gamaleya Research Center of Epidemiology and Microbiology, Moscow, Russia; ^2^Chumakov Federal Scientific Center for Research and Development of Immune-and-Biological Products of Russian Academy of Sciences, Moscow, Russia; ^3^Department of Pathology, Riga Stradins University, Riga, Latvia; ^4^Latvian Biomedical Research and Study Centre, Riga, Latvia; ^5^Engelhardt Institute of Molecular Biology, Russian Academy of Sciences, Moscow, Russia; ^6^National Medical Research Center for Tuberculosis and Infectious Diseases, Moscow, Russia; ^7^Department of Microbiology, Tumor and Cell Biology, Karolinska Institutet, Stockholm, Sweden; ^8^Chemistry Department and Belozersky Institute of Physico-Chemical Biology, Lomonosov Moscow State University, Moscow, Russia; ^9^Science for Life Laboratory, Stockholm University, Stockholm, Sweden; ^10^Russian State Center for Quality and Standardization of Veterinary Drugs and Feed (VGNKI), Moscow, Russia; ^11^Evrogen, Moscow, Russia; ^12^Sechenov First Moscow State Medical University, Moscow, Russia

## Abstract

HIV-induced immune suppression results in the high prevalence of HIV/AIDS-associated malignancies including Kaposi sarcoma, non-Hodgkin lymphoma, and cervical cancer. HIV-infected people are also at an increased risk of “non-AIDS-defining” malignancies not directly linked to immune suppression but associated with viral infections. Their incidence is increasing despite successful antiretroviral therapy. The mechanism behind this phenomenon remains unclear. Here, we obtained daughter clones of murine mammary gland adenocarcinoma 4T1luc2 cells expressing consensus reverse transcriptase of HIV-1 subtype A FSU_A strain (RT_A) with and without primary mutations of drug resistance. In *in vitro* tests, mutations of resistance to nucleoside inhibitors K65R/M184V reduced the polymerase, and to nonnucleoside inhibitors K103N/G190S, the RNase H activities of RT_A. Expression of these RT_A variants in 4T1luc2 cells led to increased production of the reactive oxygen species (ROS), lipid peroxidation, enhanced cell motility in the wound healing assay, and upregulation of expression of *Vimentin* and *Twist*. These properties, particularly, the expression of *Twist*, correlated with the levels of expression RT_A and/or the production of ROS. When implanted into syngeneic BALB/C mice, 4T1luc2 cells expressing nonmutated RT_A demonstrated enhanced rate of tumor growth and increased metastatic activity, dependent on the level of expression of RT_A and *Twist*. No enhancement was observed for the clones expressing mutated RT_A variants. Plausible mechanisms are discussed involving differential interactions of mutated and nonmutated RTs with its cellular partners involved in the regulation of ROS. This study establishes links between the expression of HIV-1 RT, production of ROS, induction of EMT, and enhanced propagation of RT-expressing tumor cells. Such scenario can be proposed as one of the mechanisms of HIV-induced/enhanced carcinogenesis not associated with immune suppression.

## 1. Introduction

HIV-induced depletion of CD4^+^ T-helper cells determines high prevalence of HIV/AIDS-associated malignancies, including Kaposi sarcoma, non-Hodgkin lymphoma, and cervical cancer. In the era of antiretroviral therapy (ART), their rates have sharply declined but still remain elevated many fold compared to the general population. HIV-infected people also have increased risks for the forms of cancer not directly associated with the immune suppression, such as lung cancer, liver cancer caused by infections with hepatitis B and hepatitis C viruses, and anogenital and oropharyngeal cancer associated with human papillomavirus (HPV) infections [1]. Multiple studies reported significant increases in their rates [2–5].

The exact mechanisms of HIV-induced carcinogenesis under the successful ART are not known; however, a series of studies show the direct role of HIV, or rather of HIV proteins, in cancer progression. HIV-1 matrix protein p17 promotes B-cell growth in non-Hodgkin lymphoma by modifying intracellular signaling and promoting genomic instability leading to cell transformation; this occurs also in HIV transgenic mice [6, 7]. Furthermore, p17 generates a prolymphangiogenic microenvironment predisposing the lymph nodes to lymphoma growth and metastasis [8], increasing the aggressiveness of human triple-negative breast cancer cells [9]. HIV-1 Nef promotes angiogenesis and tumorigenesis, synergizing with Kaposi's sarcoma (KS) KSHV oncoprotein K1 [10, 11]. Mechanistically, Nef inhibits the apoptotic function of p53; it decreases the half-life of p53 and interferes with p53 DNA binding activity and transcriptional activation [12]. HIV-1 glycoprotein gp120 stimulates glycolysis [13]. Increased glycolysis, also known as the Warburg effect, is a well-known feature of the majority of tumors that supports unconstrained proliferation and invasion of tumor cells [14, 15]. Indeed, gp120 has been shown to promote proliferation, migration, and survival of tumor cells when expressed on the viral particles, on the surface of infected cells, or as a virus-free soluble protein [13, 16]. Expression of HIV-1 Tat has been associated with the development of B-cell lymphomas [17, 18] and colorectal [19] and HPV-associated cancers [20]. Tat inhibits epithelial cytodifferentiation, blocks apoptosis, increases cell migration/motility, and accelerates tumor formation [19]. In tumor cells, it induces a significant reduction in the expression of cell cycle inhibitors of transcription and an increase in the levels of proliferation markers [21] and stimulates growth of tumor cells [22]. Thus, both structural and regulatory HIV-1 proteins demonstrate direct carcinogenic effects and/or promote the effects of known carcinogens.

Exposure of the oral keratinocytes from HIV-negative individuals to individual HIV proteins (Tat and gp120) alone or in the virions induces epithelial mesenchymal transition (EMT). Furthermore, introduction of Tat into the human uterine cervical carcinoma cells causes an upregulation of expression of HPV oncoprotein E6 with concomitant decrease in the levels of p53 [23]. Keratinocytes affected by HIV proteins (but not the unexposed ones) can then be transformed by HPV-16 DNA, exhibiting loss of cell adhesion and increased proliferation and migration/mobility critical for the progression of neoplastic processes [24]. This led to the suggestion that the promotion of EMT in the urogenital mucosa driven by HIV proteins could be one of the mechanisms by which HIV-1 enhance the carcinogenic effect of HPV oncoproteins [24].

Interestingly, a panel of HIV proteins, including ones mentioned above, has been shown to induce the production of reactive oxygen species (ROS) [18, 25–27]. ROS as such are weak carcinogens, but strong tumor promoters [28]; chronic overproduction of ROS leads to the spontaneous tumor formation [29], indicating that the induction of ROS might underlie the direct carcinogenicity of HIV-1.

We have previously found that the transient expression of HIV-1 reverse transcriptase (RT) in the mammalian cells induces the production of ROS, oxidative stress, and oxidative stress response [26, 27]. The biological implications of these findings (except for a negative effect of ROS production on the cellular immunogenicity of RT following DNA immunization) remained unknown. Here, we have explored the effects of RT-induced ROS production on the growth and motility/migration of tumor cells *in vitro* and *in vivo* on the model of murine mammary gland adenocarcinoma 4T1luc2 cells made to express a panel of HIV-1 RT variants. We found that stable expression of RT leads to an increase in the production of ROS above the already high levels observed in the parental tumor cells. RT-expressing cells exhibit enhanced migration (motility) and a shift to a mesenchymal phenotype, concomitant with an increased expression of the transcription factors Twist and Snail, which coordinate EMT. In syngeneic immunocompetent mice, these properties of RT-expressing cells lead to the enhanced tumor growth and increased metastatic activity. We found the above features to correlate with the expression of RT and/or the production of ROS. Analysis of the complex events induced by the expression of a single HIV-1 protein, the reverse transcriptase, advances our understanding of the possible mechanism(s) of HIV-driven carcinogenesis unrelated to immune suppression.

## 2. Materials and Methods

### 2.1. Design of the Consensus RT of HIV-1 Subtype A FSU-A Strain

The full-length sequences of the reverse transcriptase (RT) of the variants of HIV-1 subtype A FSU_A strain isolated from the treatment-naïve patients on the territory of the former Soviet Union were selected from the HIV sequence database (http://www.hiv.lanl.gov/content/index) and Stanford Drug Resistance database (*n* = 44) (Suppl. [Supplementary-material supplementary-material-1]). The following sequences were used, designated by HIV subtype, country of collection, year of collection, and GenBank accession number of HIV isolate: A1.GE.1999.99GEMZ011.DQ207944; A1.KZ.2002.02KZKAR300435.EF589042; A1.KZ.2002.02KZPAV300480.EF589043; A1.KZ.2002.02KZPAV300497.EF589039; A1.KZ.2002.02KZPAV300502.EF589044; A1.KZ.2002.02KZYUZ300413.EF589040; A1.KZ.2002.02KZYUZ300425.EF589041; A1.RU.2000.RU00051.EF545108; A1.RU.2002.RU01029.JQ292892; A1.RU.2003.03RU20_06_13.AY500393; A1.RU.2005.RU_560_1125_JA.JQ292895; A1.RU.2006.RU_915_1016.JQ292896; A1.RU.2006.RU_915_1035.JQ292897; A1.RU.2006.RU_915_1038.JQ292898; A1.RU.2006.RU_915_1041.JQ292899; A1.RU.2006.RU_SP_B_049.JQ292900; A1.RU.2007.Irkutsk_5.JQ292891; A1.RU.2008.DEMA108RU003.KF716491; A1.RU.2008.DEMA108RU004.KF716492; A1.RU.2008.PokA1Ru.FJ864679; A1.RU.2008.RUA001.JQ292893; A1.RU.2008.RUA007.JQ292894; A1.RU.2010.10RU6617.JX500696; A1.RU.2010.10RU6792.JX500695; A1.RU.2011.11RU6950.JX500694; A1.UA.2000.98UA0116.AF413987; A1.UA.2001.01UADN121.DQ823358; A1.UA.2001.01UADN139.DQ823357; A1.UA.2001.01UAKV254.DQ823361; A1.UA.2001.01UAOD10.DQ823365; A1.UA.2001.01UAOD35.DQ823366; A1.UA.2001.01UAOD89.DQ823367; A1.UA.2001.01UAPol293.DQ823359; A1.UA.2001.01UAPol294.DQ823356; A1.UA.2001.01UAPol303.DQ823360; A1.UZ.2002.02UZ0659.AY829209; A1.UZ.2002.02UZ0663.AY829210; A1.UZ.2002.02UZ0667.AY829211; A1.UZ.2002.02UZ0672.AY829212; A1.UZ.2002.02UZ652.AY829203; A1.UZ.2002.02UZ694.AY829205; A1.UZ.2002.02UZ698.AY829206; A1.UZ.2002.02UZ740.AY829208; A1.BY.2013.KT983615.

Sequences were aligned using Multiple Sequence Comparison by Log-Expectation (MUSCLE; http://www.ebi.ac.uk/Tools/msa/muscle/), and consensus sequence was generated with Geneious 8.1.2 software (Biomatters Ltd., Auckland, New Zealand, https://www.geneious.com/academic/). Amino acids in variable positions of the consensus sequences were chosen with the help of covariance networks obtained by squaring the difference between the number of observed and expected amino acid pairs and normalizing this difference by the number of entries (excluding gaps) in each column (the observed minus expected squared method, OMES) using custom written scripts kindly provided by Prof. J. Tavis and Dr. M. Donlin from St. Louis Medical School, USA [30]. A humanized synthetic gene encoding the corresponding amino acid sequence was designed using the web service utility at http://genomes.urv.es/OPTIMIZER [31] and the online customer portal at http://www.invitrogen.com. To ensure adequate protein expression, the expression-optimized gene was provided with an AAT-ATG-GGA sequence fused to its 5′-end. This resulted in the extension of the N-terminal region of RT with additional amino acids Met-Gly. The resulting mRNA was checked for the absence of undesirable folding (UNAFold at http://mfold.rna.albany.edu/ and OPTIMIZER at http://genomes.urv.es/OPTIMIZER/). The coding sequence for the consensus HIV-1 RT_A (RT_A) was synthesized by Evrogen (Moscow, Russia).

### 2.2. Cloning of RT_A Gene for Prokaryotic Expression and Generation of Plasmids Expressing RT_A Variants

RT_A-encoding DNA was cloned into the plasmid p6HRT-prot (a gift from Dr. S. Le Grice, NCI-Frederick, Frederick, MD) in substitution for the sequence encoding the wild-type p66/p51 heterodimeric RT of HIV-1 HXB2 strain. Plasmid p6HRT-prot had two restriction sites for BamHI endonuclease at the 5′-end of the polynucleotide sequence encoding RT of HIV-1 HXB2 (RT_B), one of them was deleted by site-directed mutagenesis. RT_A encoding sequence was cloned between the remaining 5′-BamHI and 3′-SalI restriction sites, generating p6HRT_A encoding the consensus RT of HIV-1 FSU_A1 strain with the N-terminal hexahistidine tag. Mutations M184V and K65R conferring resistance to nucleoside RT inhibitors (NRTI) and K103N and G190S conferring resistance to nonnucleoside RT inhibitors (NNRTI) were introduced into p6HRT_A by site-directed mutagenesis (Evrogen) generating plasmids p6HRT_An (65/184) and p6HRT_Ann (103/190), respectively.

### 2.3. Reverse Transcriptase Expression and Purification

Heterodimeric p66/p51 RT_A variants were expressed in M-15 (pREP4) *E. coli* cells (Qiagen, Hilden, Germany) transformed by the plasmids p6HRT_A, p6HRT_An (65/184), and p6HRT_Ann (103/190) and purified as was described previously [32]. In brief, cells harboring the expression vector were grown overnight in 5 mL of LB medium supplemented with 10 g/L glucose, 150 mg/L ampicillin (A150), and 50 mg/L kanamycin (K15) at 37°C. After the overnight culture, cells were harvested by centrifugation, the pellet was resuspended in 300 mL of the fresh medium supplemented with A150 and K50, and cells were grown at 37°C up to OD_600_ 0.5. At this point, isopropyl-*β*-D-thiogalactopyranoside (IPTG) was added to a final concentration of 1 mM, and cells were grown for additional 5 h and then harvested by centrifugation at 4000g at 4°C for 30 min. The cell pellet was resuspended on ice in 15 mL of buffer A (25 mM Tris-HCl, pH 7.9, 500 mM NaCl, 10% (*v*/*v*) glycerol, 5 mM *β*-mercaptoethanol) with 1% (*v*/*v*) Triton X-100 supplemented with protease inhibitors phenylmethylsulfonyl fluoride (PMSF, 1 mM) and Protease Inhibitor Cocktail for expression of hexahistidine-tagged proteins (Sigma, Darmstadt, Germany). The suspension was lysed by sonication on ice and pelleted at 8000g for 30 min. The clarified lysate was applied onto a 2 mL Ni-NTA-agarose column (Novagen, Darmstadt, Germany). The column was successively washed with buffer A containing 10, 30, and 40 mM imidazole (10 column volumes each); protein was eluted by the same buffer containing 200 mM imidazole. Fractions (0.7 mL each) were analyzed by the Bradford protein assay [33]. The target fractions were pooled, dialyzed against buffer B (25 mM Tris-HCl (pH 7.5), 300 mM NaCl, 10% (*v*/*v*) glycerol, 5 mM DTT, and 10 mM MgCl_2_) for 2 hours at 4°C, then dialyzed against buffer C (25 mM Tris-HCl (pH 7.5), 300 mM NaCl, 50% (*v*/*v*) glycerol, 5 mM DTT, and 10 mM MgCl_2_) for 12 hours at 4°C, aliquoted, and stored at −20°C.

### 2.4. Polymerase Activity Assays

The RT assays using activated DNA (Amersham, Buckinghamshire, UK) were performed as follows: the standard reaction mixture (20 *μ*L) contained 0.75 *μ*g of activated DNA, 0.02–0.05 *μ*g RT, 3 *μ*M dATP, 30 *μ*M of dCTP, dGTP, and dTTP, 1 *μ*Ci [*α*-^32^P] dATP in a Tris-HCl buffer (50 mM, pH 7.5) supplemented with 10 mM MgCl_2_, and 0.2 M KCl. The reaction mixtures were incubated for 12 minutes at 37°C and applied onto Whatman 3MM filters with 0.5 M EDTA solution to stop the reaction. After drying on air, the filters were washed twice with 10% trichloroacetic acid, then twice with 5% trichloroacetic acid, once with ethanol, and dried on air. The radioactivity was measured by the Cherenkov method [34] in a Tri-Carb 2810 TR scintillation counter (PerkinElmer, Franklin Lakes, NJ, USA).

Kinetic parameters of the DNA-dependent DNA polymerase reaction included the Michaelis constant (*K*_M_) determined as the substrate concentration at half of the maximal velocity and maximal reaction velocity (*V*_max_) determined as the velocity, which cannot be further increased by the increase of the substrate concentration. Kinetic parameters were determined by the standard methods using RT variants in concentrations corresponding to 17 nM of RT of HIV-1 HXB2 strain (RT_B), expressed and purified as described previously [32]. 20 *μ*L of the reaction mixture contained dATP in varying concentrations (0.05, 0.07, 0.11, 0.25, 0.5, and 1.5 *μ*M); other components of the reaction mixture and reaction conditions were as described above. *K*_M_ to dATP and *V*_max_ were calculated using Sigma Plot 8.0 software (Systat Software Inc., London, UK).

### 2.5. RNase H Activity Assay

RNase H activity of HIV-1 RTs was tested by using 6.7 mmol of 18-ribo-Fl/18-deoxy duplex formed by 18-mer oligoribonucleotide 18-ribo-Fl 5′-r(GAUCUGAGCCUGGGAGCU)-fluorescein-3′ and 18-mer oligodeoxyribonucleotide 5′-d(AGCTCCCAGGCTCAGAUC)-3′. RNA/DNA duplex was added to the reaction mixtures consisting of 15 *μ*L of 50 mM Tris-HCl pH 8.0 containing 60 mM KCl, 10 mM MgCl_2_, and various concentrations of RT_A variants or RT_B (5, 20, 100, and 400 nM) and incubated for 15 min at 37°C. The reaction was stopped by adding 80 *μ*L of a solution containing 7 mM EDTA, 0.375 M sodium acetate, 10 mM Tris-HCl, pH 8.0, and 0.125 mg/mL glycogen. The mixture was extracted by phenol/chloroform, and RNA/DNA fragments were precipitated with ethanol. The reaction products were separated by electrophoresis in a 20% polyacrylamide/7 M urea gel. Gel images were recorded using a phosphoimager and then quantified using Quantity One 4.6.6. (Bio-Rad, USA). In order to determine the specific RNase H activity of the recombinant RT, 100 *μ*L reaction mixtures containing 100 *μ*M RNA/DNA duplex, 50 mМ Tris-HCl pH 8.0, 10 mM MgCl_2_, 60 mM KCl and RTs in variable concentrations (20, 40, and 80 nM) were incubated at 37°C and aliquots of 15 *μ*L were removed after 1, 1.5, 2.5, 3.5, 5.5, and 15 minutes and quenched with 80 *μ*L of stop solution (1 mM CH_3_COONa, 200 mM glycogen, and 200 mM EDTA). RNA/DNA fragments were precipitated with ethanol at 0°C and analyzed by electrophoresis in a 20% polyacrylamide/7 M urea gel. The specific activities of enzymes as dependence of the initial velocities of RNase H reaction on the concentration of the enzyme were calculated as described previously [35, 36]. Kinetic parameters of the ribonuclease reaction were determined by standard methods using RT variants in different concentrations conferred to their specific activities: 40 nM of RT_B variant, 45.6 nM of RT_A, 41.6 nM of RT_An variant, and 56.8 nM of RT_Ann variant. 10 *μ*L of the reaction mixture composed of RT and RNA/DNA duplex in varying concentrations (100, 110, 125, 150, 200, 250, 500, 1000, and 2500 nM) dissolved in the buffer containing 50 mМ Tris-HCl (pH 8.0), 10 mM MgCl_2_, and 60 mM KCl was incubated at 37°C for 3 minutes and then analyzed as described above. *K*_M_ and *V*_max_ parameters were calculated using Sigma Plot 8.0 software (Systat Software Inc., London, UK).

### 2.6. Preparation of Lentiviral Vectors Encoding RT Variants

Coding sequences for RT_A, RT_An, and RT_Ann were recloned from p6HRT_A, p6HRT_An (65/184), and p6HRT_Ann (103/190) into the lentiviral vector pRRLSIN.cPPT.PGK (Addgene plasmid #12252; a gift from D. Trono) generating a set of lentiviral vectors pLV-RT_A, pLV-RT_An, or pLV-RT_Ann (Suppl. [Supplementary-material supplementary-material-1]). Lentiviral particles were produced by transient transfection of 293T cells as described elsewhere [37] and concentrated 10-fold with Amicon Ultra-15 100K centrifuge concentrators (Merck-Millipore, Darmstadt, Germany). Infectious titers of the lentiviral particles were determined on HT1080 cells by quantitative real-time PCR [37] using standard samples of HT-1080 DNA with a known number of viral genome copies.

### 2.7. Lentiviral Transduction of 4T1luc2 Cells and Isolation of Clones Expressing RT_A Variants

Lentivirus preparations were used to transduce murine mammary gland adenocarcinoma cells expressing firefly luciferase 4T1luc2 (PerkinElmer). Transduction of 4T1luc2 cells was performed with the multiplicity of lentiviral infection of 1, 5, and 20 of transducing units per cell for nonmutated and 1 and 10 for mutated RT_A variants. The resulting cell lines were cloned to single cells by limiting dilution in 96-well plates generating monoclonal populations of 4T1luc2 derivative clones. All 4T1luc2 derivatives were cultured in the full RPMI-1640 medium with 10% FBS and 100 mg/mL penicillin/streptomycin mix at 37°C in 5% CO_2_ and split every 2-3 days.

The presence of the sequences encoding RT_A variants in the genome of 4T1luc2 derivative clones was confirmed by PCR with a pair of primers specific for the lentivector backbone and flanking the RT insert (Suppl. [Supplementary-material supplementary-material-1], Suppl. [Supplementary-material supplementary-material-1]).

Doubling time of the derivative clones was estimated as described previously [38, 39].

### 2.8. Confirmation of RT Expression by RT-PCR and Western Blotting

RNA was isolated using TRI reagent (Sigma, Darmstadt, Germany) according to the manufacturer protocol and reverse transcribed using MINT reverse transcription kit (Evrogen). Presence of RT mRNA was confirmed using conventional PCR with primers, specific to *RT* and *HPRT1* (hypoxanthine-guanine phosphoribosyltransferase) (Suppl. [Supplementary-material supplementary-material-1]); PCR products were resolved by agarose electrophoresis.

Expression of RT_A variants was analyzed by Western blot of cell lysates performed as described previously [40]. The exact amount of each RT variant produced per cell was calculated using protein calibration curve built using recombinant RT, resolved by PAGE and Western blotting using polyclonal anti-RT antibodies. These antibodies are equally effective in the detection of different RT variants [41]. Signals from the bands containing known amount of recombinant RT were quantified by ImageJ software (http://rsb.info.nih.gov/ij) and used to build a calibration curve. Using this curve, we assessed the amount of RT in the aliquots of cell lysates loaded onto the gel, and by dividing this value by the number of cells used to prepare the lysate, we determined the levels of RT expression per cell for each derivative clone. In all further assays of the activities of RT_A variants, we assessed their effect in a known number of cells, calculated the effect per cell, and then normalized the effect per cell to the amount of RT variant produced by this cell.

### 2.9. Assessment of the Production of Reactive Oxygen Species (ROS)

Production of ROS was registered with 2′,7′-dichlorodihydrofluoresceine diacetate (DCFH2-DA) as described previously [42, 43]. Cells treated with oxidants *tert*-butylhydroquinone (tBHQ) (Sigma, Darmstadt, Germany) and H_2_O_2_ at 100 *μ*M concentrations were used as positive controls of ROS induction. Antioxidants N-acetyl cysteine (NAC) in concentration 5 mM were used as a general inhibitor of ROS production, [44], and tocopherol (both from Sigma, Darmstadt, Germany) in concentration 1 *μ*M, as the inhibitor of lipid peroxidation [45].

### 2.10. Analysis of Lipid Peroxidation

The level of malondialdehyde (MDA) in the cells was determined using high-performance liquid chromatography with mass-spectrometric detection. A 50 *μ*L aliquot of cell suspension containing known number of cells was supplemented with 250 *μ*L of 1% sulfuric acid. The mixture was shaken vigorously for 1 min by hand, then vortexed for 2 min to disrupt the cells, and supplemented with 300 *μ*L of 1.3 mM DNPH (Acros Organics, New Jersey, USA). The mixture was kept in a dark place at the ambient temperature for 40 min to derivatize MDA. After the completion of the reaction, the mixture was neutralized by adding 20 *μ*L of 4 M NaOH. The sample was centrifuged at 10000g for 15 min at 4°C, and the supernatant was collected and transferred into an HPLC vial. Samples, 5 *μ*L each, were injected into an HPLC system with 150∗2 mm Pursuit XRs 5 *μ*m C18 column (Agilent, Santa Clara, USA). The analysis was carried out in the gradient mode using 0.5% formic acid solution as phase A and 0.5% formic acid solution in methanol as phase B. MDA-DNPH signal was recorded by 6500 QTRAP mass-spectrometer (Sciex, Framingham, USA) using three MRM channels: 235 > 159 (quantifier), 235 > 189, and 235 > 143 (qualifiers). The linear calibration curve was set by the acid hydrolysis of tetramethoxypropane (Cell Biolabs, San Diego, USA) in 1% sulfuric acid using five standard points (10–200 pmol/mL). NIH3T3 cells served as a negative and serial dilutions of a solution containing known concentrations of MDA (MyBioSource, Vancouver, Canada) as positive controls. The MDA quantity was calculated per cell and normalized to the amount of RT variant produced by this cell (calculated as described above).

### 2.11. Wound Healing Assay

Cells were harvested in 24-well cell culture plates to obtain absolutely confluent monolayer in standard medium or in the medium with 5 mM N-acetyl cysteine. A cross-shaped wound was scratched at the center of each well with a 200 *μ*L sterile tip. Wells were photographed at 0, 13, and 18 hours using a light microscope (Leica, Wetzlar, Germany). Borders of the gaps were marked using the Adobe Photoshop CC software (Adobe, San Jose, CA, USA). Distances between the edges were measured using the ImageJ software.

### 2.12. Isolation of Nucleic Acids, Reverse Transcription, and Semiquantitative PCR

Cell culture medium was discarded; cells were detached using 0.25% Trypsin with EDTA before lysis in AmpliSens Riboprep lysis buffer (CRIE, Moscow, Russia). Nucleic acids were isolated using AmpliSens Riboprep kit (CRIE, Moscow, Russia) according to the manufacturer's instructions and reverse transcribed using AmpliSens Reverta-FL (CRIE, Moscow, Russia). Gene-specific PCRs were performed on a RotorGene 6000 (Qiagen, Hilden, Germany) cycler using primers specific to E-cadherin, N-cadherin, Vimentin, Twist, and Snail genes (Suppl. [Supplementary-material supplementary-material-1]) and SYBR Green (Thermo Fisher Scientific, Waltham, MA USA). Levels of mRNAs were measured relative to *HPRT1* mRNA used as a reference. Relative gene expression levels were calculated using the ddCt method [46].

### 2.13. Assessment of Tumorigenicity of 4T1luc2 Derivative Clones

Animal experiments were performed in the animal facilities of the NF Gamaleya Research Center of Epidemiology and Microbiology (GRCEM), Ministry of Health of the Russian Federation (Moscow, Russia) and of the Latvian Biomedical Research and Study Centre (Riga, Latvia). The experiments were carried in compliance with the bioethical principles adopted by the European Convention for the Protection of Vertebrate Animals Used for Experimental and Other Scientific Purposes (Strasbourg, 1986). Experimental procedures were approved by the ethical committee of the GRCEM (protocol N10, March 14, 2017) and by the Latvian Animal Protection Ethics Committee of the Latvian Food and Veterinary Service (permit no. 99 from April 4, 2018). Eight-week-old BALB/c mice from “Pushchino” breeding facility of the Institute of Bioorganic Chemistry RAS (Pushchino, Russia) or Laboratory Animal Center University of Tartu (Tartu, Estonia) were housed under a 12 h/12 h light-dark cycle with *ad libitum* access to water and food. Mice were marked with earmarking and housed in groups of three to five animals per cage. During all interventions, mice were anesthetized with the 1 L/min flow of air containing 4% isoflurane at the induction and 2.5% at the maintenance stage, administered through the facial masks.

The capacity of the derivative cell lines to form tumors and metastases was tested by ectopic implantation into 8-week-old female BALB/c mice. Prior to injection, 4T1luc2 and derivative clones grown in the selective medium were detached, sedimented, washed with serum-free RPMI-1640, stained for viability with Trypan Blue dye (Life Technologies, Carlsbad, CA), then counted in a hemocytometer, and aliquoted 10^4^, 2 × 10^4^, and 4 × 10^4^ cells in 50 *μ*L of RPMI-1640 in sterile test tubes. Aliquots were injected with a 25G needle mounted on an insulin syringe (B Braun, Germany) subcutaneously into two sites, to the right and to the left of the base of the tail. Tumor size was assessed by morphometric measurements done at regular intervals using calipers; tumor volume was calculated using the standard formula for xenograft volume [47, 48]: *V* = *xy*^2^/2. Tumor growth was also assessed by bioluminescent imaging (BLI) on Spectrum CT (PerkinElmer, Waltham, MA, USA) following the procedures recommended by PerkinElmer and described by us earlier [27]. Monitoring of bioluminescence was done directly after the implantation, then on days 1, 2, 4, and 6 and then every 2-3 days until the tumor volume of the first mouse in the experiment reached the volume of 1 cm^3^ which was set as the experimental endpoint. Mice were weighed at each monitoring point. At the experimental endpoint, normally on days 18 to 21 after the implantation, mice were humanely euthanized and organs were collected. All experiments were done in two independent runs, 1^st^ on five and 2^nd^ time on three mice per cell line and cell dose.

### 2.14. *In Vivo* Bioluminescence Imaging

Detailed procedure of *in vivo* imaging was described earlier [27]. In brief, freshly prepared solution of XenoLight D-luciferin potassium salt (PerkinElmer, Waltham, MA, USA) was administered into mice in the amount of 150 mg/kg based on their actual weight. The substrate was diluted in 200 *μ*L PBS and injected intraperitoneally. After 10 min, mice were anesthetized as described above. The area of the skin over the tumor in the radius of 1.5 cm was shaved and depilated using a depilating cream for sensitive skin. After that, mice were transferred into the In Vivo Imaging System (IVIS; IVIS Spectrum CT, PerkinElmer) with the continuous flow of 1 L per min of air containing 4.0% isoflurane at the initiation and 2.5% at the maintenance stages. IVIS was set to automatic exposure time (60 sec at early stages, reduced to 2 to 10 sec at late stages of tumor growth). Images were captured, and total photon flux from the regions of interest (ROI) of the same area was analyzed using Living Image 4.5 Software® (PerkinElmer). After the completion of monitoring, mice were transferred back to their cages with close placement to reduce the risk of hypothermia.

### 2.15. Experimental Endpoint and Collection of Mouse Organs for Rapid *Ex Vivo* Assessment of the Formation of Metastases

At the experimental endpoint, mice were anaesthetized as described above. Freshly prepared solution of XenoLight D-luciferin potassium salt (PerkinElmer) in PBS was injected into mice intraperitoneally in an amount of 150 mg/kg based on the actual weight. Ten minutes later, mice were euthanized by cervical dislocation by a skilled animal handler. The fur and skin of the mice were disinfected with 70% ethanol. After that, tumors, spleen, liver, and lungs, affected by the distant metastasis in the 4T1 tumor model [49, 50], were dissected with surgical scissors. Tumors and organs were transferred into the wells of a 24-well tissue culture test plate (Wallac, Turku, Finland) containing 2 mL RPMI-1640 medium. *Ex vivo* bioluminescent imaging of tumors and organs was performed as described for *in vivo* imaging. Thereafter, tumors and all organs, except for spleens, were transferred to 5 mL of 4% formaldehyde solution in PBS, incubated for 24 to 48 h at +6°C, then washed five times with PBS, and used to prepare FFPE blocks, according to the standard protocol [51].

### 2.16. Preparation of Splenocytes and Analysis of Their Immune Reactivity

Spleens were washed from luciferin with PBS, transferred into the Petri dishes containing 3 mL RPMI, and grinded with a syringe plunger, and single-cell splenocyte cultures were prepared as described earlier [52]. Population of white blood cells in the splenocyte samples was analyzed on a FACSAria II cytometer (BD Biosciences, USA). Data were exported as FCS3.0 files with the use of FACSuite software and analyzed using FlowJo X.07 program (FlowJo LLC, Ashland, USA). General lymphocyte and granulocyte areas were defined on forward scatter (FSC) versus side scatter (SSC) plot. Remaining splenocytes were tested for the capacity to proliferate in response to stimulation with synthetic peptides. Cytokine production by stimulated cells was assessed using IFN-*γ*/IL-2 Fluorospot Kits (Mabtech, Nacka Strand, Sweden) following the procedures described earlier [52]. In brief, single-cell cultures 2.5 × 10^5^ splenocytes per well in the duplicate wells were stimulated for 18 h with a peptide representing the region between aa 145 and 168 of RT_A QYNVLPQGWKGSPSIFQSSMTKIL, which we have shown to contain a cluster of CD4^+^ and CD8^+^ T cell epitopes recognized by CD4^+^ and CD8^+^ T cells of BALB/c mice (S Petkov, M Isaguliants, unpublished data) and a peptide GFQSMYTFV derived from firefly luciferase (both peptides from Synpeptide Co. LTD, Shanghai, China). Peptides were dissolved in PBS and used at the final concentration of 10 *μ*g/mL. Unrelated peptides and medium alone served as negative and mitogen Concanavalin A (5 *μ*g/mL) as a positive control. The stimuli were diluted in the complete culture media consisting of RPMI supplemented with 5% FBS, 100 U/mL penicillin, 100 *μ*g/mL streptomycin, and 0.3 mg/mL glutamine (Gibco, Thermo Fisher, Waltham, MA, USA). After 20 h incubation, plates were processed following the protocol provided by the manufacturer and read on ImmunoSpot CTL S5 UV Analyzer (Cellular Technology Limited (CTL), Cleveland, USA) to assess the number of splenocytes producing IFN-*γ* and IL-2 (cytokine-producing cells; SFCs) per well. The number of cytokine-producing cells was recalculated per 10^6^ splenocytes.

### 2.17. Tumor Histology and *Ex Vivo* Assessment of the Metastases

FFPE blocks were prepared from the formalin-fixed tumor tissues and murine lungs and livers and sectioned on cryostat microtome according to the standard protocols (https://www.protocolsonline.com/histology/sample-preparation/paraffin-processing-of-tissue/). Sections mounted on slides were dewaxed, rehydrated, stained with Mayer's hematoxylin solution, then washed, rinsed, and counterstained with eosin Y solution, after that, dehydrated, washed with absolute alcohol, and covered with cover slips for microscopic evaluation. Histological evaluation was based on the standard parameters such as acinar formation, nuclear size, and pleomorphism and mitotic activity [51]. Grade of the tumors was calculated according to [53]. The slides were examined by light microscopy (Leica DM500, Wetzlar, Germany). Formalin-fixed, Paraplast-embedded liver tissues were used to diagnose and evaluate the formation of metastases. For each mouse, the area of tumor metastases was quantified in 25 high-power (×400) microscope fields of hematoxylin-eosin-stained slides by computer-assisted morphometry using specialized NIS-Elements software (Nikon, Tokyo, Japan). Total DNA was isolated from five freshly prepared sections using Allprep DNA/RNA FFPE Kit (Qiagen). The presence of the sequences encoding RT_A variants was confirmed by PCR using a pair of primers, one specific for the lentivector backbone and the other to RT coding sequence (Suppl. [Supplementary-material supplementary-material-1]).

### 2.18. Statistics

Statistical analysis was performed using STATISTICA AXA 10.0 (StatSoft Inc., OK, USA). Nonparametric statistics were chosen as appropriate for sample sizes < 100 entries. Continuous but not normally distributed variables, such as enzymatic parameters, the normalized levels of gene expression, the total photon flux, tumor volume, number and size of the metastases, and the number of cytokine-producing cells (SFCs), were compared in groups using Dunn's test for pairwise multiple comparisons of the ranked data as the post hoc test following Kruskal-Wallis test and pairwise by Mann-Whitney *U* test or Fisher LSD tests. Data grouped by two variables were analyzed using ordinary two-way ANOVA with the correction for multiple comparison. Correlation analysis was done using Spearman Rank Correlation test. *R* in Spearman test represented the regular Pearson product-moment correlation coefficient (Pearson *r*) in terms of the proportion of variability accounted for computed from the ranks. Spearman *R* assumed that the variables under consideration were measured on at least an ordinal (rank order) scale with the individual observations (cases) ranked into two ordered series. Multiple regression analysis was done by first building and then analyzing the correlation matrix; all calculations involved in the actual multiple regression analysis were performed in double precision. Matrix inversion was accomplished via sweeping. The regression weights, residual sums of squares, tolerances, and partial correlations were calculated as part of the sweeping operation. The *F* value (Regression Mean Square/Residual Mean Square), *t* (df), and resulting *p* value were used as an overall *F* test of the relationship between the dependent variable and the set in independent variables. *F* value associated with the multiple *R* and the *t* values associated with the regression coefficients were calculated using standard built-in formulas (STATISTICA AXA 10.0). Parametrical statistical analysis was performed using IBM SPSSv23 software including descriptive assessment as detection of mean values and standard deviation (SD). For all types of analysis, *p* values < 0.05 were considered significant.

## 3. Results

### 3.1. Design of the Consensus RT of HIV-1 Subtype A FSU_A Strain and Its Variants with Compromised Polymerase and RNase H Activities

We have earlier found that transient expression of RT of HIV-1 clade B strains in eukaryotic cells induces oxidative stress [26, 27]. Here, we aimed to determine if this is a general property of HIV-1 RTs, whether or not it depends on the enzymatic activities of RT, and what are the *in vitro* and *in vivo* implications of the induction of oxidative stress for RT-expressing cells. To resolve these questions, we designed a consensus RT of a different clade, namely, HIV-1 clade A FSU_A strain and a panel of its variants with compromised polymerase and RNase H activities.

**Figure 1:**
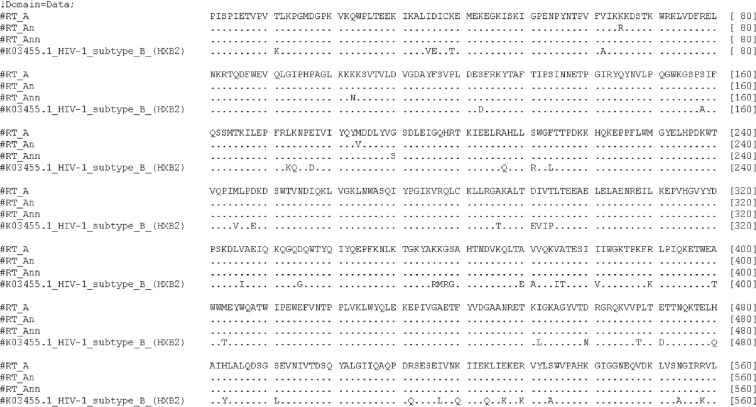
Alignment of the amino acid sequences of the consensus HIV-1 reverse transcriptase and its drug resistance variants designed in the study. “RT_A,” consensus of amino acid sequences (*n* = 44) of HIV-1 subtype A FSU_A strain isolated between 1999 and 2012, with amino acids in variable positions chosen using OMES-based covariance network. “RT_An,” RT_A variant with introduced mutations of resistance to NRTI K65R and M184V; “RT_Ann,” RT_A variant with introduced mutations of resistance to NNRTI K103N and G190S; “K03455.1 HIV-1 subtype B (HXB2),” reference sequence of HIV-1 subtype B RT used for position numbering [63].

Amino acid sequences of RTs of HIV-1 FSU_A isolates from the treatment-naïve patients without known drug resistance mutations were selected from HIV-1 sequence database and HIV Drug Resistance database (*n* = 44). The majority of sequences dated 1999 and 2012 were from the Russian Federation (41%) and the rest from other countries of the former Soviet Union: Ukraine (23%), Uzbekistan (18%), Kazakhstan (14%), and Belarus and Georgia (2% each) (Suppl. [Supplementary-material supplementary-material-1]). In concordance with the earlier observations [54], we observed very low average amino acid diversity of RT amino acid sequences, which allowed us to build a reliable consensus sequence of HIV-1 FSU_A RT (RT_A) (Figure 1).

On the basis of this consensus, we designed mutant RT variants with compromised activities of polymerase and RNase H. This was achieved by the insertion of biologically relevant primary mutations of drug resistance to nucleoside (NRTI) and nonnucleoside (NNRTI) inhibitors characteristic to FSU_A isolates: mutations of resistance to NRTI are known to reduce the polymerase, and to NNRTI, both the polymerase and RNase activities of the enzyme [55]. The most frequent mutation of resistance to NRTI worldwide is M184V/I, followed by K65R/N, L74V/I, Y115F, and Q151M, and the most prevalent for FSU_A is M184V, followed by K65R/N [56–58]. These mutations diminish the polymerase activity of RT [59]. The most frequent primary mutation of resistance to NNRTI worldwide is G190S (http://hivdb.stanford.edu/DR/NNRTIResiNote.html). It is also the most frequent mutation of resistance to NNRTI in patients infected with HIV-1 FSU_A [58, 60]. It is present either alone or in combination with other mutations, such as K103N, and is never detected in HIV-1 patients naïve to treatment with NNRTIs. G190S and K103N have little effect on RT-driven polymerization but selectively slow down RNase H cleavage and delay initiation of DNA synthesis, resulting in a significant reduction of viral replication competence [61, 62]. Based on this, we designed RT_A with M184V and K65R/N (RT_An) and RT_A with K103N and G190S (RT_Ann) which we predicted to have reduced polymerase and RNase H activities, respectively.

Synthetic DNA encoding consensus RT_A optimized for eukaryotic expression was cloned into the prokaryotic expression vector p6HRT to generate plasmid p6HRT_A for prokaryotic expression of RT_A. Mutations M184V/K65R or K103N/G190S were introduced into p6HRT_A by site-directed mutagenesis, yielding plasmids for prokaryotic expression of RT_An and RT_Ann, respectively, to purify respective proteins and determine their enzymatic activities.

### 3.2. Prokaryotic Expression of HIV Clade A RTs and Assessment of Their Enzymatic Activity

Prokaryotic expression vectors p6HRT_A encoding RT_A, p6HRT_An (65/184) encoding RT_An, and p6HRT_Ann (103/190) encoding RT_Ann were used to transform *E. coli*. RT_A, RT_An, and RT_Ann proteins which were purified using Ni-NTA-agarose chromatography and assessed for their polymerase and RNase H activities.

The polymerase activity was assessed using a template-primer complex represented by a nicked double-stranded DNA duplex (“activated DNA”). All three RT_A variants were highly active and exhibited similar level of activity with Michaelis constant (*K*_M_) of approximately 0.4 *μ*M and maximum velocity (*V*_max_) ranging from 4 to 7.5 *μ*M/sec∗mg, comparable to RT of HIV-1 clade B HXB2 strain (Table 1). Consensus RT_A had higher *V*_max_ than RT of HIV-1 HXB2. As expected, NRTI resistance mutations led to a decrease of the polymerization rate compared to *V*_max_ of the parental RT_A (*p* = 0.005) and of NNRTI-resistant RT_Ann (*p* = 0.006; Table 1). Thus, as predicted, mutations of resistance to NRTI reduced, and to NNRTI, had no effect on polymerization rate.

Next, we assessed RNase H activity of RT_A variants using as substrate the RNA/DNA heteroduplex consisting of 18 nt DNA strand and 18 nt fluorescein-labeled RNA. All RT variants were enzymatically active even at concentrations as low as 5 nM (Suppl. [Supplementary-material supplementary-material-1]). At 5 nM concentration, RT_A variants were less active than RT_B, but at higher enzyme concentrations (100 and 400 nM), their RNase H activities were similar (Suppl. [Supplementary-material supplementary-material-1]). All three RT_A variants had lower specific activity compared to HXB2 RT (Suppl. [Supplementary-material supplementary-material-1], Table 1). As expected, the consensus RT_A and RT_An had similar specific RNase H activities, whereas RT_Ann exhibited only 60% of the activity of the consensus RT_A (Table 1). Further, we determined *K*_M_ and *V*_max_ of the RNase H reaction. All RT_A variants had lower *V*_max_ of the cleavage of RNA/DNA heteroduplex than RT of HIV-1 HXB2 (*p* < 0.05 for all pairwise comparisons; Fisher LSD test) with RT_Ann exhibiting somewhat lower *V*_max_ than RT_A (*p* = 0.09; Table 1). Thus, as predicted, mutations of resistance to NRTI had no effect, and to NNRTI, reduced the activity of RNase H. Thus, we generated two RT_A variants with deficiencies in each of the enzymatic activities, to further relate them to the ability of these enzymes to induce oxidative stress.

### 3.3. Derivatives of 4T1luc2 Cell Line Stably Expressing RT Variants

To assess the capacity of RT_A variants to generate oxidative stress and relate this to their tumorigenic potential, we generated RT-expressing derivatives of murine mammary gland adenocarcinoma 4T1 cells which form solid tumors with a high metastatic potential in BALB/c mice [64, 65]. Stable expression of luciferase reporter (Luc) in the daughter cell line 4T1luc2 (PerkinElmer, Franklin Lakes, NJ, USA) allows *in vivo* monitoring of tumor growth and metastasis formation. As we have previously shown, Luc expression induces an anti-Luc response that limits the metastatic potential of 4T1luc2 compared to the parental 4T1 cells [49]. Here, we have conducted the experiments to see if expression of RT_A variants would induce oxidative stress and through this modify the tumorigenic and metastatic potential of 4T1luc2 cells *in vitro* and *in vivo*.

Nucleotide sequences encoding RT_A, RT_An, and RT_Ann were recloned into the lentiviral vector under the control of the human phosphoglycerate kinase promoter, generating derivative vectors pLV-RT-A, pLV-RT-An, and pLV-RT-Ann, further used to transduce 4T1luc2 cells. Through this, we generated seven daughter clones of 4T1luc2 with genomic insertions of 2000 nt fragments encoding RT_A, RT_An, and RT_Ann (confirmed by sequencing) (Table 2).

**Figure 2:**
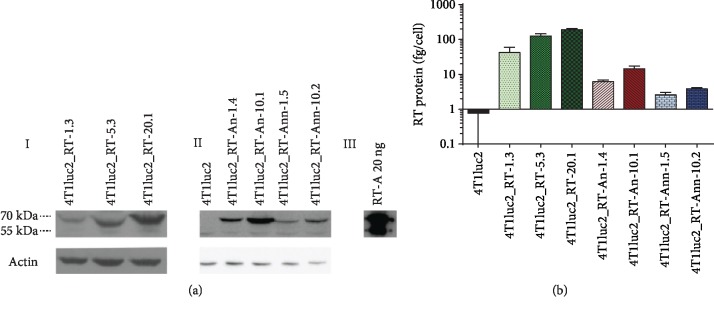
Expression of reverse transcriptase (RT) by 4T1luc2-derived cell lines carrying genomic inserts of sequences encoding variants of the consensus HIV-1 FSU_A RT. (a) Western blot analysis of lysates of derivative 4T1luc2 clones (Table 2) stained with rabbit polyclonal anti-RT antibodies [68] (I and II upper panels), and anti-actin monoclonal antibodies (I and II lower panels), recombinant RT_A as positive control (III). The parental 4T1luc2 cell line was used as a reference. Polyclonal anti-RT antibodies weakly bind to reverse transcriptase expressed by the lentivirus used to generate the parental 4T1luc2 cell line (II). Position of the weight mass marker is shown on the left. (b) Quantification of RT_A expression using ImageJ software. Signal in the lane corresponding to a given derivative clone was quantified using calibration curve built with the help of recombinant RT_A. Total amount of the expressed RT_A variant was divided by the number of cells used to make the lysate (see Materials and Methods for description). Data represent the results (mean ± SD) from three independent experiments.

All derivative clones were shown to express proteins with the expected molecular mass of 66 kDa, corresponding to the nonprocessed form of HIV-1 reverse transcriptase, specifically recognized by anti-RT antibodies (Figure 2(a)). We also detected minor amounts of the protein with molecular mass of 51 kDa corresponding to the processed form of HIV-1 RT, resulting from the cleavage of p66 by cellular proteases [66]. RT p66 forms homodimers [67], which predominate over the heterodimeric p51/p66 form (as was previously shown for RT-expressing HeLa cells [52]).

We assessed the levels of expression of RT_A variants and defined the amount of protein expressed by each of the derivative clones per one cell (Figure 2(a); see Materials and Methods for description). RT_A was expressed in the amount of 40 to 200 fg; RT_An, 6 to 10 fg; and RT_Ann, 3 to 4 fg per cell (Figure 2(b)). The level of expression of RT_A variants correlated with the multiplicity of infection (MOI) used to generate the respective clone (clones generated at higher MOI exhibited higher levels of RT expression) (Figure 2). The level of expression of RT_A, even at the lowest MOI, was higher than the level of expression of the mutated RT_A variants (Figure 2(b)).

### 3.4. RT_A-Expressing 4T1luc2 Cells Produce High Levels of Reactive Oxygen Species (ROS)

Cancer cells produce elevated levels of ROS due to high metabolic activity; modified cellular signaling; peroxisomal activity; mitochondrial dysfunction; activation of oncogenes; increased enzymatic activity of oxidases, cyclooxygenases, lipoxygenases, and thymidine phosphorylases; and ROS-mediated signaling supporting cancer survival, angiogenesis, and progression [69]. Therefore, we expected the parental 4T1luc2 cells to release increased levels of ROS and inquired if expression of RT_A variants could additionally increase ROS production and if the levels of ROS depend on the dose and nature of the expressed RT variants.

**Figure 3:**
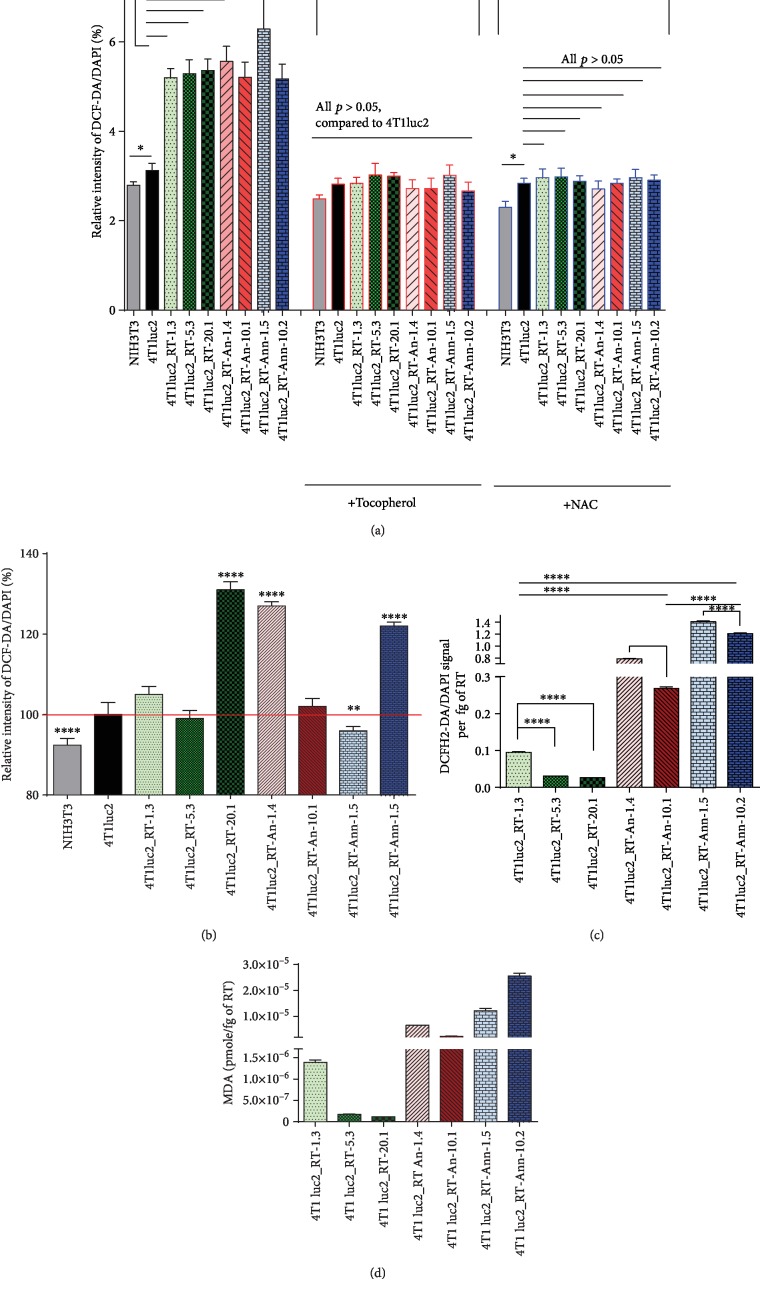
Derivatives of 4T1luc2 cells expressing variants of consensus HIV-1 FSU_A reverse transcriptase (Table 2) exhibit increased levels of ROS production (a–c) and lipid peroxidation (d). ROS production was measured using fluorescent dye DCFH2-DA and normalized to the signal generated by DAPI staining. Total levels of ROS per cell on day 6 of cell culture; production of ROS is quenched by treatment with antioxidants tocopherol (1 *μ*M) and NAC (5 mM); red asterisk indicates significant differences between 4T1luc2_RT-Ann-1.5 and other RT-expressing variants (*p* < 0.05) (a). Total levels of ROS production per cell on day 14 of cell culture relative to the levels exhibited by the parental 4T1luc2 cells (b). ROS levels on day 14 of cell culture normalized to the amount of RT_A variant (in fg) produced by one cell (c). Level of lipid peroxidation on day 14 of cell culture assessed as the concentration of MDA in the lysate of a single cell normalized to the amount of RT_A variant (in fg) produced by one cell (d). Values represent mean ± SD from two independent assays run in duplicates. ns: not significant; ^∗^*p* < 0.05; ^∗∗^*p* < 0.01; ^∗∗∗^*p* < 0.001; ^∗∗∗∗^*p* < 0.0001 (Kruskal-Wallis, followed by Mann-Whitney tests or ordinary one-way ANOVA followed by unpaired *t*-test).

Levels of ROS production in 4T1luc2 derivative clones were assessed with the help of the sensor fluorescent dye 2′,7′-dichlorodihydrofluoresceine diacetate (DCFH2-DA). DCFH2-DA reacts with various types of ROS yielding a fluorescent product which allows to assess the general redox status of cells [70]. Early after seeding, the parental 4T1luc2 cell line demonstrated increased total levels of ROS per cell compared to immortal murine cells (line NIH3T3; Figure 3(a)). Levels of ROS in cell lines expressing RT_A variants exceeded the level in the parental cell line by nearly 100% (Figure 3(a)). Treatment with tocopherol which prevents peroxide oxidation, or NAC which neutralizes free radicals, decreased the levels of ROS to the levels observed in the parental 4T1luc2 cells (Figure 3(a)). The effect of expression of RT_A variants on 4T1luc2 cells was comparable to that of oxidants H_2_O_2_ or tBHQ (Suppl. Fig. [Supplementary-material supplementary-material-1]). Interestingly, addition of H_2_O_2_ or tBHQ to RT_A-expressing 4T1luc2 cells induced no further increase in ROS production, while antioxidant treatment reduced ROS production to basal levels (Suppl. Fig. [Supplementary-material supplementary-material-1]). After two weeks of culturing, all 4T1luc2 daughter clones reduced the levels of ROS production but still produced more ROS per cell than the immortal NIH3T3 cells (Figure 3(b)). Levels of ROS exhibited by RT-expressing clones tended to correlate with the level of RT expression per cell (*R* = 0.53; *p* = 0.05, Spearman ranking test). Thus, stable expression of RT_A variants led to an increase in ROS production over the already high levels characteristic to murine adenocarcinoma cells. Interestingly, during prolonged cell culture, some of the cell lines were able to quench it, at least partially (Figure 3(b)).

We wanted to see if longitudinal ROS production was related to the level of RT expression and/or to the nature of RT_A variant. For this, we normalized the levels of ROS in expressing cells after two weeks in culture to the average amount of RT_A variant per cell produced by this cell line (Figure 3(c)). We found that cells expressing nonmutated RT_A were able to significantly quench ROS production; quenching was less efficient in cells expressing RT_An and inefficient on cells expressing RT_Ann (*p* < 0.05; Figure 3(c)). High levels of ROS in a cell maintained over a long period of time were translated into increased levels of lipid peroxidation (expressed as relative levels of MDA per fg of RT; Figure 3(d)). Levels of MDA were highly correlated to the levels of ROS (*R* = 0.83; *p* = 0.0008). Production of MDA was reduced by treatment of cells with antioxidants (Suppl. [Supplementary-material supplementary-material-1]). Thus, constitutive expression of RT_A variants caused an increase in the production of ROS over the levels characteristic to the parental adenocarcinoma cells, resulting, on the long term, in increased levels of lipid peroxidation. Interestingly, however, this effect was minimal for cells expressing high levels of nonmutated RT_A (Figure 3(d)).

### 3.5. RT_A-Expressing 4T1luc2 Cells Exhibit High Motility in the Wound Healing Assay

Next, we assessed if expression of RT_A variants influences the motility of 4T1luc2 cells in the wound healing assay. All derivative clones had similar doubling time of 13 to 15 hours. Considering this, cells grown on plates were assessed for the capacity to heal the gap within 14 hours.

**Figure 4:**
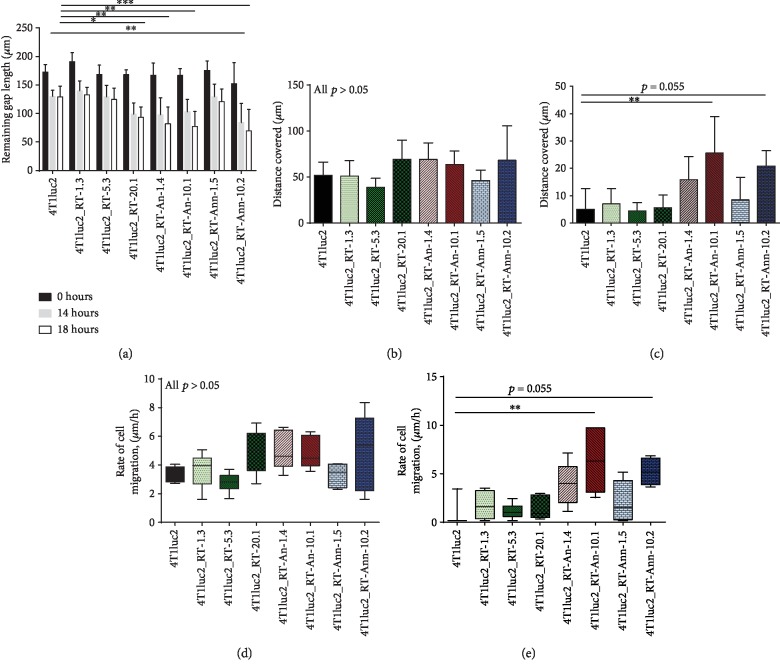
Performance of the derivatives of 4T1luc2 cells expressing variants of consensus HIV-1 FSU_A reverse transcriptase (Table 2) in the wound healing assay. (a) The length of the gap (wound) in *μ*m, at the time of the scratch and 14 and 18 hours later. (b) Distance covered by the cells during the first 14 hours after the scratch, calculated as a difference in the length of the gap at the time of the scratch and after 14 hours. (c) Distance covered by the cells in the interval between 14 and 18 hours after the scratch. (d) Speed of cell migration during the first 14 hours after the scratch, calculated as the covered distance divided by 14 h. (e) Speed of cell migration in the interval between 14 and 18 hours after the scratch. The length of the gap (distance covered by the cells) was calculated based on the light microscopy images using ImageJ software. All values are expressed as mean ± SD from five independent experiment runs. ns: not significant; ^∗^*p* < 0.05; ^∗∗^*p* < 0.01; ^∗∗∗^*p* < 0.001; ^∗∗∗∗^*p* < 0.0001.

After the scratch (Suppl. [Supplementary-material supplementary-material-1], panels 1, 2), we observed a difference between the parental 4T1luc2 cell line and derivative clones in the closure of the wound during the first 14 hours (a tendency for 4T1luc2_RT-20.1, 4T1luc2_RT-An-1.4, and 4T1luc2_RT-An-10.1 and significant difference for 4T1luc2_RT-Ann-10.2; Figure 4(a); *p* < 0.05). Overall, the derivative clones healed larger areas than the parental 4T1luc2 clone, with a higher speed of wound healing (Figures 4(b) and 4(d)). We also tested if the derivative clones were capable of continued growth after passing the doubling time (with a short distance left to cover between the edges of the healing wound). For this, we measured wound healing 18 hours after the scratch (Suppl. [Supplementary-material supplementary-material-1]; panels 1, 3). Interestingly, while the parental cell line and clones expressing nonmutated RT_A nearly stopped growing (*p* > 0.05 in all pairwise comparisons; RT_1.3, 5.3, and 20.1 behaved similarly to the parental 4T1luc2 cells; Figure 4(c)), cells of four clones expressing mutant RT_As continued to move towards each other, albeit with a different speed (Figure 4(e)). We noticed that cells of these clones were moving both as a monolayer (epithelial phenotype) and as single cells (mesenchymal phenotype) (exemplified by Suppl. [Supplementary-material supplementary-material-1]).

Thus, the expression of RT_A variants led to an enhanced capacity of the derivative 4T1luc2 clones to heal the wound. The rates of cell migration during the first 14 hours after the scratch (Figure 4(d)) directly correlated to the total level of ROS produced per cell in longitudinal culture (Figure 3(b); *R* = 0.775, *p* < 0.024, Spearman test) but not to the level of RT expression.

### 3.6. RT_A-Expressing 4T1luc2 Cells Express Increased Levels of Factors Associated with the Epithelial Mesenchymal Transition (EMT)

**Figure 5:**
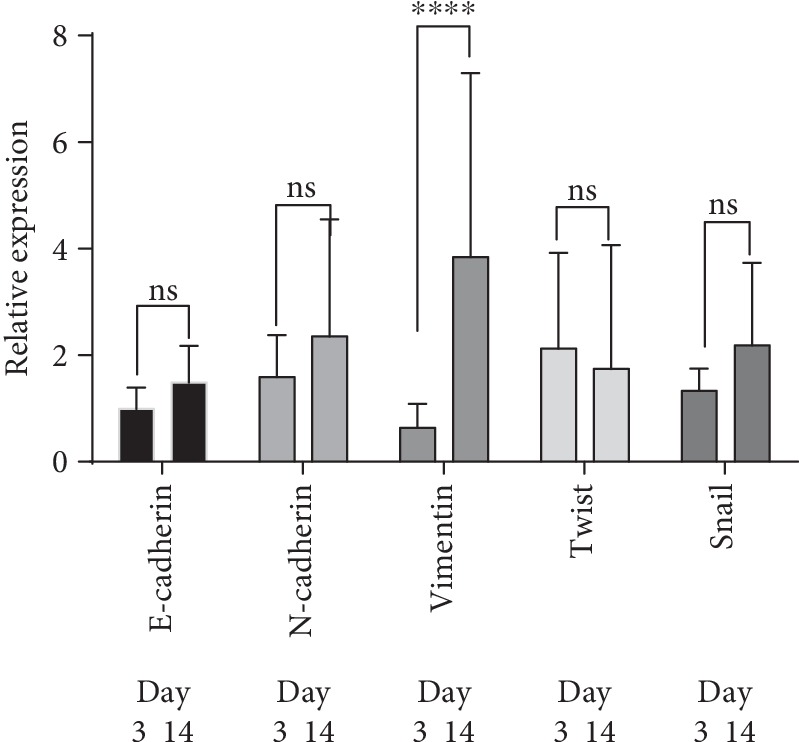
Relative expression levels of EMT markers in the derivatives of 4T1luc2 cells expressing variants of consensus HIV-1 FSU_A reverse transcriptase (Table 2) after 3 and 14 days in culture. Expression level was normalized to *HPRT1* expression and calculated as fold change compared to the parental 4T1luc2 line. Results are presented as mean ± SD from two independent assays run in duplicates. ns: not significant; ^∗∗∗∗^*p* < 0.0001 by the ordinary two-way ANOVA.

Acceleration of growth at the late stages of wound healing and appearance of “jumping” cells may indicate a change in the cell phenotype. This stimulated us to investigate the expression in these cells of the factors associated with epithelial/mesenchymal transition (EMT). Expression of the basic factors indicative of the epithelial or mesenchymal cell phenotype, *E-cadherin* as characteristic of the epithelial and *N-cadherin*, *Vimentin*, and transcription factors *Twist* and *Snail* as characteristic of the mesenchymal cell phenotype [71, 72], was assessed by the levels of respective mRNAs normalized to that in the parental 4T1luc2 cells. The assessment was done at days 3 and 14 of cell culture. All markers demonstrated stable levels of expression, except for *Vimentin*, expression of which significantly increased with time in all cell lines (*p* < 0.0001). This demonstrated that in their phenotype all clones expressing RT_A variants were similar to the parental cells but during long culture (14 days equivalent to 24 doubling times) acquired more pronounced mesenchymal features (Figure 5, Suppl. [Supplementary-material supplementary-material-1]).

**Figure 6:**
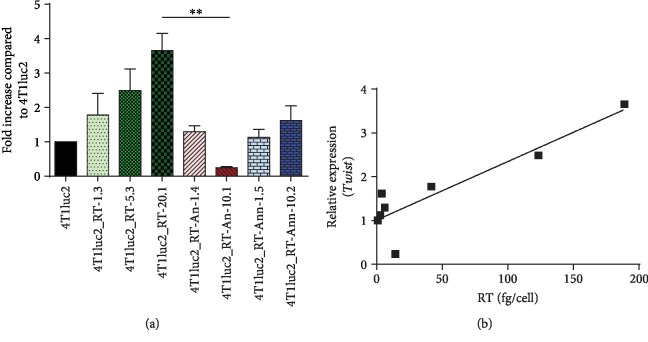
Relative levels of expression of *Twist* by the derivatives of 4T1luc2 cells expressing variants of consensus HIV-1 FSU_A reverse transcriptase (Table 2) after 14 days in culture (a) and correlation of the relative expression of *Twist* with the levels of expression of RT_A variants (b). Expression level of *Twist* was normalized to the expression of *HPRT1* and calculated as fold change compared to the levels exhibited by the parental 4T1luc2 cells. Values are presented as mean ± SD from the assay run in triplicates. The amount of RT_A variant produced per cell and expression of *Twist* at day 14 were correlated using Spearman ranking test (*R* = 0.9, *p* = 0.002); ^∗∗^*p* < 0.01.

We have further assessed if expression of EMT markers after 3 and 14 days in culture correlated with the levels of expression of RT_A variants (Suppl. [Supplementary-material supplementary-material-1]). None of the parameters showed any correlation with the level of expression of RT_A variants (*E-cadherin*: *R* = −0.3, *p* = 0.5; *N-cadherin*: *R* = 0.21, *p* = 0.7; *Vimentin*: *R* = 0.4, *p* = 0.4; *Snail*: *R* = 0.2, *p* = 0.7, Spearman test), except for *Twist*; the latter reached high significance by day 14 (*R* = 0.9, *p* = 0.002) (Figure 6; Suppl. [Supplementary-material supplementary-material-1]). Levels of Twist expression on day 14 showed a weak tendency to correlate to the levels of ROS (*R* = 0.71, *p* = 0.13; Spearman test). We confirmed ROS dependence of the upregulation of expression of *Twist* by treating cells with the antioxidant NAC. NAC treatment resulted in a complete disappearance of the correlation between the levels of RT expression and observed relative levels of *Twist* mRNA (Suppl. Fig. [Supplementary-material supplementary-material-1]).

*Twist* expression correlated with the levels of expression of *Snail* (*R* = 0.78, *p* = 0.02) and *N-cadherin* (*R* = 0.8, *p* = 0.016) and tended to correlate with expression of *Vimentin* (*p* = 0.11) (all on day 14 of culture) (Suppl. Fig. [Supplementary-material supplementary-material-1]). Importantly, early expression of *N-cadherin* and late expression on *Vimentin* highly correlated with the production of ROS (*p* = 0.02, Suppl. [Supplementary-material supplementary-material-1]). This pointed at the interconnection between oxidative stress and induction of factors associated with EMT: *N-cadherin*, *Vimentin*, and *Twist*. Interestingly, relative levels of *Twist* expression on day 14 together with the levels of ROS and the level of RT expression reliably predicted the speed of migration of RT-expressing cells in the wound healing assay (multiple regression analysis, Suppl. [Supplementary-material supplementary-material-1]). The dependence of the motility on the level of expression of RT was the least significant, but still important for the prediction. Thus, stable expression of RT_A variants led to a shift towards mesenchymal phenotype with enhanced expression of *Twist*, which governs the late stages of epithelial mesenchymal transition, which we have shown to depend on the production of ROS.

### 3.7. Tumorigenicity of 4T1luc2 Derivative Clones Expressing Variants of RT_A

**Figure 7:**
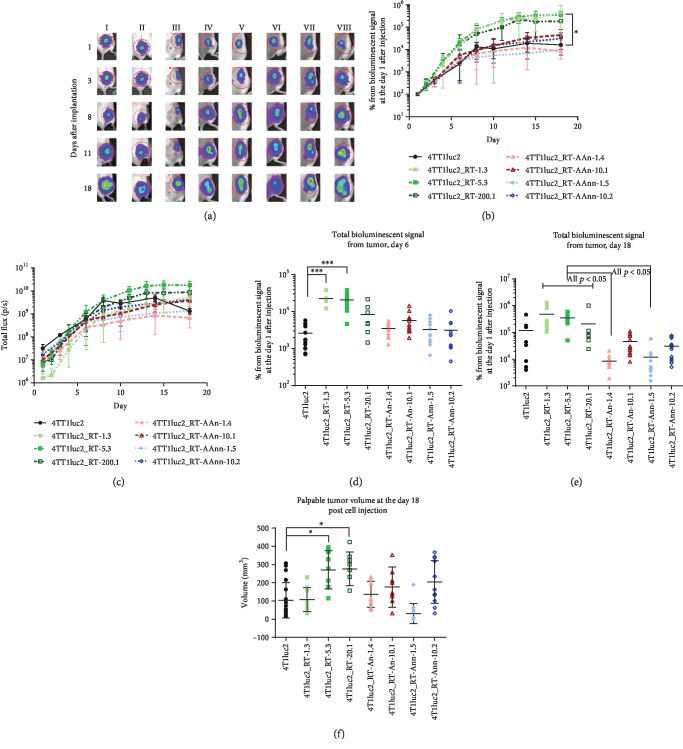
Formation of solid tumors by the derivative clones of 4T1luc2 expressing variants of HIV-1 FSU_A reverse transcriptase (RT_A) after ectopic implantation into BALB/c mice. *In vivo* bioluminescent images showing growth of representative tumors formed by clones 4T1luc2_RT-1.3 (I), 4T1luc2_RT-5.3 (II), 4T1luc2_RT-20.1 (III), 4T1luc2_RT-An-1.4 (IV), 4T1luc2_RT-An-10.1 (V), 4T1luc2_RT-Ann-1.5 (VI), 4T1luc2_RT-Ann-10.2 (VII), and parental cells 4T1luc2 (VIII); red circles show the regions of interest (ROI) from which the total luminescent signal was collected (a). Growth of tumors assessed as the average total photon flux from the sites of injection encircled by ROI (b), the average percent change in the total photon flux compared to the photon flux from the site obtained on day 1 (c), the total photon flux from each of the injections sites on days 6 (d) and 18 (e) as percent of that on day 1, and palpable tumor size at the experimental endpoint (f). All group values represent the mean ± SD. After day 3, 4T1luc2_RT-1.3 and 4T1luc2_RT-5.3 derivative clones demonstrated significantly higher total photon flux, and 4T1luc2_RT-20.1 tended to have higher photon flux (*p* = 0.1) than the parental 4T1luc2 cells. ^∗^*p* < 0.05, ^∗∗^*p* < 0.005, ^∗∗∗^*p* < 0.0005, and ^∗∗∗∗^*p* < 0.0001, Kruskal-Wallis, followed by Mann-Whitney tests.

Finally, we performed an *in vivo* study of tumorigenic and metastatic potential of RT_A-expressing clones by implanting them into the syngeneic BALB/c mice; parental 4T1luc2 cells served as control. All RT_A-expressing derivative clones formed solid tumors within 6 to 7 days after ectopic implantation. Starting from day 3 and up to the experimental endpoint, tumors formed by cells expressing nonmutated RT_A were significantly larger than tumors formed by the parental cell line, whereas tumors formed by 4T1luc2 cells expressing mutant RT_As did not differ in size from those formed by 4T1luc2 cells (Figures 7(a) and 7(e)). Furthermore, tumors exhibited different growth rates (Figures 7(b) and 7(c)). Clones expressing RT_A demonstrated higher *in vivo* growth at the exponential phase (Figures 7(c) and 7(d)) and also by the experimental endpoint (Figure 7(e)). The *in vivo* growth characteristics of 4T1luc2 clones expressing mutant RT_As did not differ from those of the parental cells (Figures 7(a)–7(f)).

**Figure 8:**
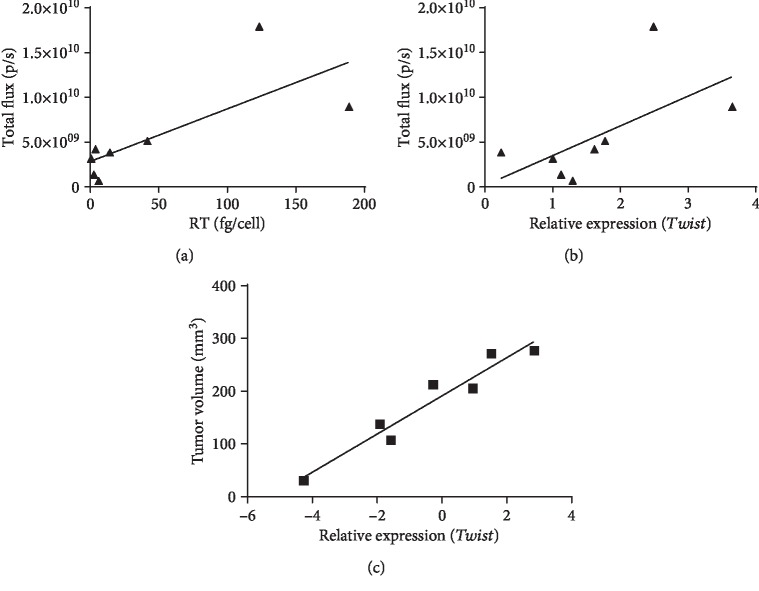
Correlations (Spearman correlation test) between the tumor sizes represented by the total photon flux from tumor on day 18 and the levels of expression of RT_A variant per cell (*R* = 0.76, *p* < 0.05) (a); the levels of expression of Twist (normalized against the expression of HPRT1 and represented as fold increase compared to that of the parental 4T1luc2 cells) (*R* = 0.74, *p* < 0.05) (b); and palpable tumor volume on day 18 and change in the levels of expression of Twist from day 3 to day 14 in % (*R* = 0.96, *p* = 0.0001) (c).

The size of the tumors generated by a given clone, as assessed by an increase in the total photon flux from day 1 to day 18, strongly correlated with the *in vitro* properties of the inducing cell line, namely, the level of late expression of *Twist* in an *in vitro* assay and the level of RT expression (Figures 8(a) and 8(b); *R* = 0.73 and *R* = 0.76 correspondingly, both *p* < 0.05, Spearman test). Tumor volume in the morphometric assay (Figure 7(f)) tended to correlate with the late expression of *Twist* (*R* = 0.74, *p* = 0.05) and strongly correlated with the increase in expression of *Twist* between days 3 and 14 of an *in vitro* culture of the respective tumor-inducing cells (*R* = 0.96, *p* = 0.0001; Figure 8(c)).

**Figure 9:**
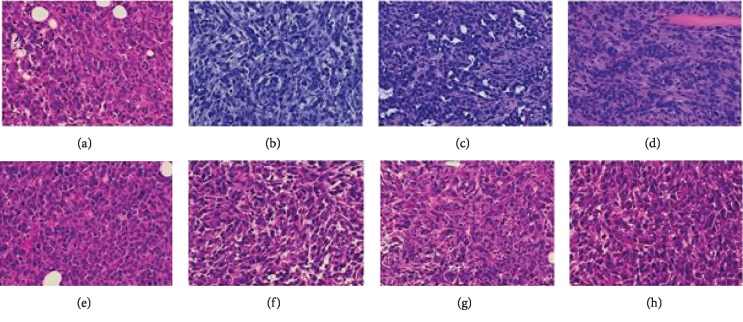
Histochemical characterization of the solid tumors formed by the parental 4T1luc2 cells (a) and their derivatives expressing variants of HIV-1 FSU_A reverse transcriptase (RT_A) after ectopic implantation into BALB/c mice: 4T1luc2_RT-1.3 (b), 4T1luc2_RT-5.3 (c), 4T1luc2_RT-20.1 (d), 4T1luc2_RT-An-1.4 (e), 4T1luc2_RT-An-10.1 (f); 4T1luc2_RT-Ann-1.5 (g), 4T1luc2_RT-Ann-10.2 (h). H&E staining: magnification ×400.

At the experimental endpoint, tumors were excised, fixed, paraffin embedded, sectioned, H&E stained, and examined by an experienced oncopathologist. All tumors were high grade (G3) poorly differentiated adenocarcinomas with increased cellular and nuclear atypia, stromal desmoplasia, and frequent necrotic areas, multifocal for tumors formed by the parental and RT_A-expressing 4T1luc2 cells or central for tumors formed by 4T1luc2 cells expressing mutant RT_As (Figures 9(a)–9(h)). Analysis of DNA isolated from the sections confirmed presence of inserts corresponding to the coding sequences of RT_A variants.

### 3.8. Metastatic Potential of 4T1luc2 Derivative Clones Expressing Variants of RT_A

**Figure 10:**
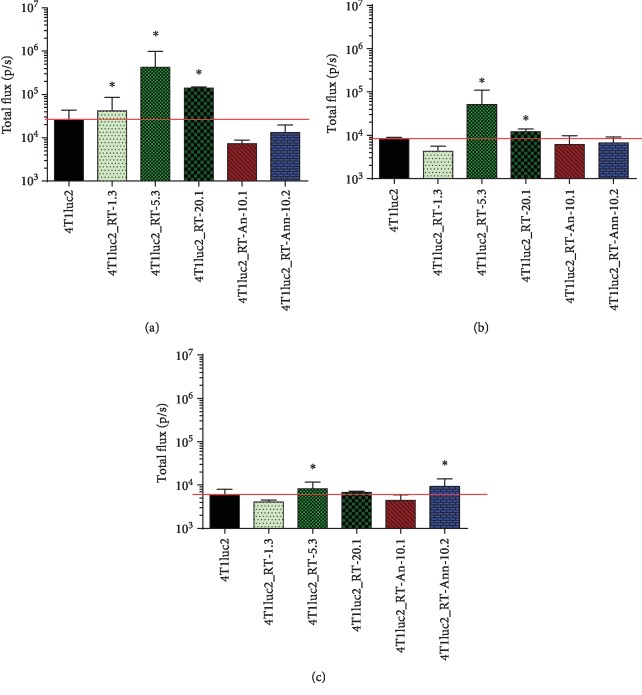
Localization of luciferase-expressing cells in the organs of mice implanted with 4Tluc2 and derivative clones expressing variants of HIV-1 FSU_A reverse transcriptase (RT_A) after ectopic implantation into BALB/c mice as assessed by *ex vivo* bioluminescent imaging of the lungs (a), liver (b), and spleen (c). The values represent the mean total flux (p/s) ± SD (*n* = 3). ^∗^Significant difference from the value exhibited by the respective organ in 4Tluc2-implanted mice (*p* < 0.05; Mann-Whitney test).

Next, we inquired if constitutive expression of HIV-1 RT variants could influence the metastatic activity of 4T1luc2 cells, in terms of the number of distant metastases and/or their organ distribution. We compared the metastatic activity of 4Tluc2 clones expressing RT_A with that of two clones expressing relatively high levels of mutant RT_A variants, 4T1luc2_RT-An-10.1 and 4T1luc2_RT-Ann-10.2. Organs (lungs, liver, and spleen) of mice bearing respective tumors were excised and immediately after assessed for the presence of luciferase-expressing cells by *ex vivo* bioluminescent imaging. Earlier, by injecting mice with known numbers of 4Tluc2 cells, we have established a direct correlation between the total photon flux from the injected tissue and the number of injected luciferase-expressing 4T1luc2 cells [73]. The total flux from the organs of mice bearing tumors expressing RT_A significantly exceeded the total flux from the organs of mice bearing tumors formed by the parental 4T1luc2 cells. This was especially pronounced for lungs and livers of mice implanted with cell lines expressing high levels of nonmutated RT_A (4Tluc2-RT-5.3 and 4Tluc2-RT-20.1; Figures 10(a) and 10(b)) indicating that these mice had significantly more luciferase-expressing cells in these organs than mice with tumors formed by the parental cell line.

This approach did not allow to establish the actual number of metastases, as they could have been formed by different numbers of cells (from as few as 3 to as many as 10000 [74]). To characterize the formation of metastases, we performed histochemical screening of the selected organs.

**Figure 11:**
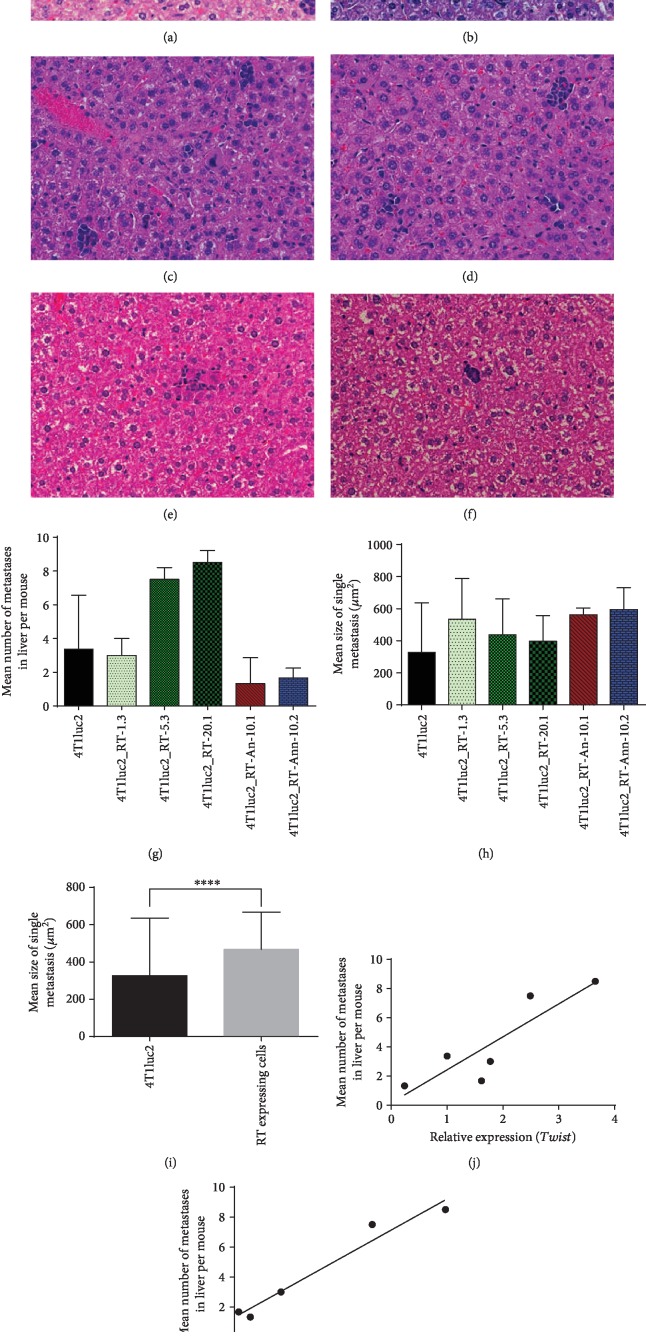
Histochemical characterization of the formation of metastases in the livers of BALB/c mice ectopically implanted with 4Tluc2 and derivative clones expressing variants of HIV-1 FSU_A reverse transcriptase (RT_A). H&E staining of liver sections of mice injected with 4T1luc2 and its RT-expressing subclones (×400): 4T1luc2 (a), 4T1luc2_RT-1.3 (b), 4T1luc2_RT-5.3 (c), 4T1luc2_RT-20.1 (d), 4T1luc2_RT-An-10.1 (e), 4T1luc2_RT-Ann-10.2 (f). Mean number (g) and size (h) of metastases. Mean size of liver metastasis formed by all RT_A-expressing clones compared to the parental 4Tluc2 cells (i). Correlations between the mean numbers of metastases in a mouse liver assessed by histochemical staining and the levels of expression by the respective 4T1luc2 clone of *Twist* (day 14 of cell culture) (j) and of RT_A variant per cell (k). Ten high-power fields per mouse (*n* = 3) were screened to assess the number of metastasis; their size was evaluated using NIS-Elements software (Nikon, Tokyo, Japan), both values were represented as mean ± SD. Expression level of *Twist* was normalized to the expression level of *HPRT1* and represented as fold change compared to the level in the parental 4T1luc2 cells. Comparisons were done using the Mann-Whitney test, and correlations, using the Spearman correlation test. ^∗^*p* < 0.05, ^∗∗^*p* < 0.005, ^∗∗∗^*p* < 0.0005, and ^∗∗∗∗^*p* < 0.0001.

Earlier, we have shown that the expression of luciferase by 4T1 cells compromise the capacity of these cells to form metastases in distant organs, specifically in the liver [49]. Based on these observations, we have chosen to assess the actual number of metastases formed by the derivatives of 4Tluc2 cells in the liver as a sensitive indicator of possible deficiencies in the metastatic activity. The assessment of H&E-stained liver sections revealed that mice implanted with 4T1luc2 cells expressing RT_A had significantly more metastases in the liver than mice implanted with the parental 4Tluc2 cells or 4Tluc2 derivatives expressing mutant RT_As (Figures 11(a)–11(g)).

The metastases formed by 4T1luc2 expressing RT_A variants did not differ in size (Figure 11(h)); however, on the average, their size was larger than the size of metastasis formed by the parental 4T1luc2 cells (Figure 11(i)). The number of metastases formed by derivative 4Tluc2 clones tended to correlate with their capacity to express *Twist* (Figure 11(j); *R* = 0.83, *p* = 0.06) and RT_A protein (per cell) (Figure 11(k); *R* = 0.9, *p* = 0.08); statistical significance was not reached due to a small sample size. No correlations were observed between the number of metastases detected by the histochemical assay and any of the *in vitro* characteristics of the clones other than *Twist* (such as the levels of production of ROS, MDA, the parameters of the wound healing assay, or the expression of EMT factors; all *p* > 0.1). Thus, expression of HIV-1 RT, particularly of the nonmutated RT_A variant, led to an increase in the metastatic activity of 4T1luc2 cells expressing the respective RT_A variant, compared to the parental cells, with the increase proportional to the expression of RT and induction of expression of *Twist*.

### 3.9. Mice Bearing Tumors Formed by the Derivatives of 4T1luc2 Cells Expressing RT_A Exhibit Splenomegaly with Granulocyte Infiltration into the Spleen and No Cellular Response to a Mitogen

**Figure 12:**
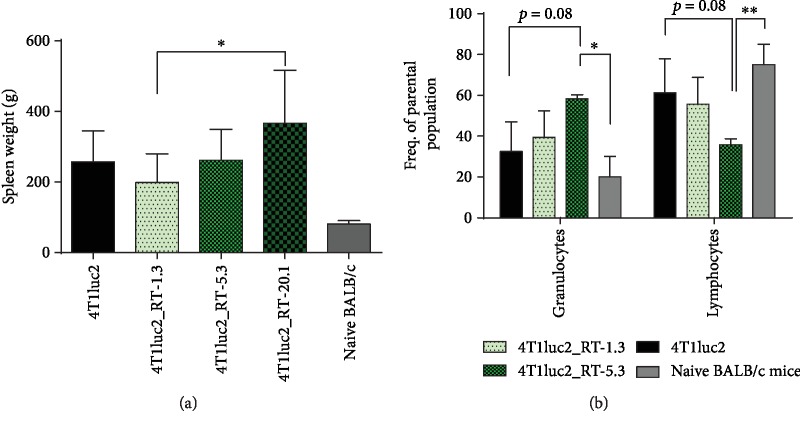
BALB/c mice ectopically implanted with 4Tluc2 and derivative clones expressing HIV-1 FSU_A reverse transcriptase (RT_A) have grossly enlarged spleens (a) and a disproportion in the leukocyte subpopulations in the spleen (b). Spleen weight, in mg (a) and proportion of leukocytes in the spleen (b) of BALB/C implanted with given 4T1luc2 derivative clones (*n* = 3-9) was assessed at the experimental end-point, and represent mean ± SD. Data from naïve BALB/c mice (*n* = 3-5) is given for comparison. ^∗^*p* < 0.05; ^∗∗^*p* < 0.005 (two-way ANOVA test).

Initial examination revealed that all mice bearing 4Tluc2-derived tumors developed splenomegaly, especially pronounced in mice bearing tumors expressing RT_A (Figure 12(a); normal spleen weight in healthy naïve 12-week BALB/c mice is 80 ± 10 mg). The weight of the spleen increased proportionally to the level of RT_A expression by the clone (not significant due to a small number of observations).

Further, we prepared single splenocyte cultures and assessed the proportion of leukocyte subpopulations in the spleens by flow cytometry. Analysis revealed a reduced number of lymphocytes and gross overrepresentation of granulocytes (Figure 12(b)), supporting the earlier findings in mice bearing 4T1 tumors [75, 76]. Due to this disproportion, splenocyte cell cultures of mice bearing RT_A-expressing tumors exhibited low proliferative response to the mitogen Concanavalin A (ConA) (given as number of IFN-*γ*-producing cells per 10^6^ splenocytes): 100 ± 50 for subclones expressing nonmutated and 1100 ± 600 for subclones expressing mutated RT_As. In mice implanted with 4T1luc2, the number of IFN-*γ*-producing cells per 10^6^ splenocytes was 400 ± 200, and in healthy mice of similar age, over 3000 IFN-*γ*-producing cells per 10^6^ splenocytes. We have also stimulated splenocytes with a peptide, which we have earlier shown to contain a cluster of CD4^+^ and CD8^+^ T cell epitopes of RT [52, 77], to see if any of them formed an immune response against RT_A as a foreign antigen. No responses were detected in any of the groups (less than 50 IFN-*γ*-producing cells per 10^6^ splenocytes). Thus, expression of HIV-1 RT by 4T1luc2 tumors induced a systemic pathological effect manifested by splenomegaly, disproportion of leukocyte populations in the murine spleen, and anergy, which was more pronounced in mice implanted with RT_A-expressing 4T1luc2 than in those implanted with the parental 4Tluc2 cells.

## 4. Discussion

Our first findings of enhanced ROS production by eukaryotic cells expressing RT of HIV-1 were made for RT of an HIV-1 clade B HXB2 strain [26, 27]. Here, we inquired if this is a general property of HIV-1 reverse transcriptases and if this property is linked to the enzymatic activity of the protein. We designed the consensus RT of HIV-1 clade A FSU_A strain (RT_A) and two RT_A variants with primary mutations of resistance to NRTI (K65R and M184V; RT_An) and NNRTI (K103N and G190S; RT_Ann) [60, 78]. NRTI resistance mutations reduce the efficacy of tRNA-primed (-)ssDNA synthesis resulting in a diminished enzyme processivity [36, 79]. NNRTI resistance mutations affect RNase H activity and DNA synthesis from tRNALys [61, 80]. By introducing these mutations, we expected to generate RT_A variants with reduced respective activities to further test how this would affect the capacity of the enzyme to induce ROS. As expected, the consensus RT_A was enzymatically active, RT-An (K65R/M184 V) was compromised in the polymerase, and RT_Ann (K103N/G190S), in the RNase H activities.

We started with the study of the effects of these RTs on an epithelial cell line. Epithelial cells appear to be grossly affected by HIV-1. Deposition of HIV-1 in the intact oropharyngeal [81], anal/rectal [82], cervicovaginal and foreskin/penile [81, 83–85], airway [86], and gastric epithelial cells [87] promotes systemic infection of CD4^+^ T lymphocytes, Langerhans/dendritic cells, and macrophages both *in vivo* and *ex vivo* [83, 84, 88–92]. This “contamination” creates proinflammatory environment characterized by the production of inflammatory mediators such as IL-6 and TNF-*α* and impairment of cell adhesion [86, 87, 90, 93]. With this in mind, we selected a cell line of epithelial origin, namely, murine mammary gland adenocarcinoma 4T1 cells syngeneic to BALB/c mice, to further evaluate the effect of RT expression on the properties of these cells *in vitro* (induction of ROS, cell phenotype, and motility) and *in vivo* (tumor growth and metastasis formation).

All variants of RT_A induced production of ROS over the already high levels exhibited by the parental 4T1luc2 cells. This supported our earlier findings for clade B RT, as ROS were induced by a panel of RT_Bs including multidrug-resistant and inactivated variants [26, 27]. The effect was long lasting in cell lines expressing low levels of RT_An and RT_Ann and subsided with time in cells overexpressing RT_A (see clones 4T1luc2_RT-5.3 and 4T1luc2_RT-20.1; Figure 3). High levels of ROS persisting over a long period of time were translated into increased levels of lipid peroxidation. Cells expressing nonmutated RT_A were able to diminish (“quench”) ROS production and prevent lipid peroxidation. “Quenching” was inefficient in cells expressing mutated RT_A variants with compromised enzymatic activities.

ROS orchestrated cell migration. The rates of cell migration directly correlated with the normalized levels of ROS produced in a prolonged cell culture (day 13; *R* = 0.9048, *p* < 0.01, Spearman test). Further, we observed a change in the pattern of cell migration in the wound healing assay, namely, clones expressing mutated “nonquenching” RT variants demonstrated steady growth with little or no contact inhibition even when cells were in close proximity (after 14 hours of culture). Cell motility could have been accelerated by ROS or ROS-induced soluble mediators released by cells residing at the edges of the wound. Indeed, it has been shown that ROS production elicits rapid ion fluxes to the cytosol and triggers the response in the adjacent cells opening them to a slow entry of extracellular ROS through aquaporins. This terminates ROS signaling in the current cell and amplifies it in the neighboring cells [94, 95]. One such soluble mediator could be H_2_O_2_ with the life of 1 ms which can cover the distances of hundreds of micrometers [96]. In support of this assumption, H_2_O_2_ at low levels is known to promote adhesion, migration, and wound healing in epithelial cells [97].

ROS play essential role in the EMT process in cancer cells by regulating extracellular matrix remodeling, cytoskeleton remodeling, cell-cell junctions, and cell mobility [98, 99]. Recent study has shown that ROS induce EMT by activating Snail expression, which then represses the expression of E-cadherin, a classical marker of the epithelial phenotype [100]. Several independent studies demonstrated that EMT can be induced by direct contact of epithelial cells with HIV proteins [24, 86, 101, 102]. To see if this is the case for 4T1luc2 cells expressing RT, we monitored them for the expression of *E-cadherin*, mesenchymal cell phenotype markers *N-cadherin* and *Vimentin*, and of two transcription factors, heavily involved in EMT, *Twist* and *Snail* [71, 98]. We compared the relative levels of expression of these EMT markers and checked if their expression was correlated with the expression of RT_A variants. Analysis revealed that at the late stages of cell culture (14 days or >20 doubling times) cell lines characterized by high levels of RT expression had proportionally increased expression of *Twist*. This was a fascinating finding considering the results of a recent study demonstrating functional linkage between *Twist* factors and ROS [103]. Based on this study, one can assume that in 4T1luc2 cells stably expressing HIV-1 RT, RT induces the production of ROS, which in its turn, directly or through mediators, upregulates the expression of *Twist* switching on the antioxidant activities of the latter. This would explain the low normalized levels of ROS (per fg of RT per cell) in 4T1luc2 daughter clones expressing high levels of nonmutated RT_A and high levels of *Twist* as a result of a “positive feedback loop”: overexpressed RT_A induces high total levels of ROS production and activates Twist, which, in its turn, suppresses the production of ROS. This would explain highly significant dependence of *Twist* expression on the level of RT expression, but not on the levels of ROS.

*Twist* occupies a key position in the EMT cascade in tumor cells, regulating the expression of a large number of genes, including *Nrf-2* [104] and *Snail* [105]. The RT/ROS-dependent induction of *Twist* described here gives additional mechanistic explanation to our earlier findings of the RT-induced upregulation of expression of Nrf-2 [26] and of *Snail* observed in this study. Overexpression of *Twist* may promote the migration of 4T1luc2 cells, as it has been previously shown, specifically in cancer cells [106, 107]. Indeed, we were able to reliably predict the motility of RT_A-expressing 4Tl cells using three parameters: the level of RT expression, expression of *Twist* on day 14, and relative levels of ROS production.

Finally, we undertook the *in vivo* study of tumorigenic and metastatic potential of RT-expressing cells compared to the parental 4T1luc2 cell line. All RT-expressing clones formed solid tumors and metastases in multiple organs. The tumors formed by 4T1luc2 clones expressing nonmutated RT_A were significantly larger than tumors formed by the parental cells or cells expressing mutant RT_A variants. Tumor size correlated with late expression of *Twist* in the *in vitro* tests and could be predicted based on the properties of implanted cells, such as the level of expression of RT and relative expression of *Twist*.

Mice harboring RT_A-expressing tumors had pronounced splenomegaly marked by infiltration of granulocytes, characteristic to the 4T1 tumor model [76]. Recent studies reported the accumulation in the tumor-bearing animals, most notably in the spleen, of immature myeloid-derived suppressor cells, neutrophils, and granulocyte-macrophage progenitor-derived splenic monocytes (CD11b^+^Gr-1^+^). These overlapping cell populations express immunosuppressive enzymes such as arginase 1 and inducible nitric oxide synthase (NOS2), produce ROS, suppress antitumor T cell activity *in vivo*, and inhibit T cell proliferation/IFN-*γ* production in coculture experiments [108]. These observations support our findings of the suppressed response of splenocytes of tumor-bearing mice to a mitogen as well as the absence of immune recognition of RT_A as foreign protein. Another nonexcluding explanation of these findings would be the properties of 4T1 cells, which release soluble mediators (detectable in conditioned media of cultured cells) inhibiting INF-*γ* production, which could also occur in the murine spleens infiltrated by the metastatic cells [109].

As the result, mice bearing 4T1luc2 tumors expressing nonmutated RT_A demonstrated complete loss of immune control of tumor growth (which may have otherwise led to the rejection of tumors [110]) and wide dissemination of metastatic luciferase-expressing cells in multiple organs, compared to that in mice bearing tumors formed by the parental 4T1luc2 cells [110]. Interestingly, mice with 4T1luc2 tumors expressing nonmutated RT_A had significantly more Luc-expressing cells in the lungs, spleen, and liver than mice implanted with cells expressing mutant RT_As. Thus, the expression of RT_A, but not of the mutated RT_A variants, “rescued” the metastatic potential of 4T1luc2 cells. This data was confirmed by the histochemical evaluation of the number of metastasis in the liver. The number of metastatic cells detected in the livers of mice bearing tumors formed by RT_A-expressing 4T1luc2 cells was significantly higher than that in mice implanted with tumor cells expressing mutated RT_As or the parental cells. The number of metastasis significantly correlated to the propensity of the inducing cells to express *Twist* in the *in vitro* assay. No correlations were observed of the number of metastatic cells (by BLI or by histology) with any other cell line parameter of the ones assessed in this study. Earlier, we had observed that 4Tluc2 cell line is impaired in the capacity to form metastases in organs and tissues other than the lungs (such as liver, brain, or bones); we attributed this property to the induction of immune response against luciferase which can clear metastatic cells expressing the reporter [49]. Here, we demonstrate that expression of nonmutated RT_A (but not of RT_An or RT_Ann) restores the metastatic potential of 4T1luc2 cells to the level of the initial 4T1 adenocarcinoma cells, possibly by increasing the level of immune suppression.

Altogether, our data on ROS induction, lipid peroxidation, cell motility, upregulation of transcription of EMT factors, specifically of *Twist*, tumor growth, and metastatic potential of 4T1luc2 cells expressing HIV-1 RT indicate that RT_A variants had differential effects on the expressing cells. Although mutated RT_A variants induced the production of ROS, they seemed to be compromised in other properties associated with malignancy, starting from inability to efficiently induce the expression of *Twist*. Thus, the *in vitro* and *in vivo* properties of RT_A-expressing cell lines were not clustered according to the upkeep of their individual enzymatic activities, either polymerase (RT_A and RT_Ann have intact polymerase activity) or RNase H (RT_A and RT_An have intact RNase H activity). In contrast, according to their properties, RT_A-expressing 4T1luc2 variants could be clustered into highly malignant clones overexpressing nonmutated RT_A and clones expressing low levels of RT_An and RT_Ann, nondistinguishable from the parental cells. In this context, it is important to mention that drug resistance mutations in RT target it to processing by the proteasome [41]. Mutations may be important not because of their effect on the enzymatic activity of the protein but as determinants of protein availability, localization, and intracellular partnerships.

Which properties of HIV-1 RT are responsible for the induction of ROS, if not the enzymatic activities, remains unknown. A review done by Warren et al. described cellular interactions of HIV-1 RT, listing factors which directly or indirectly bind to RT and regulate the reverse transcription [111]. The spectrum of RT partners included APOBEC3G, DNA Topoisomerase I, components of the Sin3a complex, GErmin2, a number of IN-binding proteins including IN1. Two of these partners are of specific interest: HuR (also referred to as ELAVL1, HuA, or MelG) and the kinase anchor protein 121 (also referred to as AKAP1 or AKAP149 as human homologue). HuR is an ubiquitously expressed 326 amino acid protein with nucleocytoplasmiс shuttling capabilities. Overexpression of HuR in cancer cells has been associated with poor prognosis and resistance to therapy. Absence of HuR is signified by the defective mitochondrial metabolism resulting in production of the large amounts of ROS and increased DNA damage [112, 113]. AKAP121 is an essential regulator of the mitochondrial respiration. Displacement of AKAP121 with an inactive analog results in the mitochondrial dysfunction manifested by the increased production of ROS [114]. Both HuR and AKAP121 are involved in the mitochondrial metabolism, their interaction with HIV-1 RT may imply their mitochondrial colocalization. Interestingly, regions of RT interacting with HuR and AKAP1 were both mapped to the RNase H domain of RT [115]. We find it reasonable to hypothesize that increased levels of ROS production observed in several RT-expressing eukaryotic cell lines tested by us previously and in RT-expressing 4T1luc2 cells tested here might be a consequence of direct interaction of HIV RT with HuR and/or AKAP121, which disturb their functions. Interference of RTs with HuR and/or AKAP121 functions would induce the production of ROS, which in its turn, would activate *Twist*, and start the cascade of EMT-related events, resulting in the enhancement of oncogenic and metastatic potential of RT-expressing tumor cells.

As we have shown earlier, drug-resistant HIV-1 RTs are proteolytically unstable with degradation governed by the proteasome. The half-life of the protein decreases from approximately 20 h for the wild-type RT to <2 hours for the multidrug-resistant enzyme forms [40, 41]. Both HuR [116] and AKAP1 [117] are degraded by the proteasome. One can speculate that binding of HuR and/or AKA121 to the mutated RT variants can target them to an enhanced proteasomal degradation. This way to deplete the pool of regulatory proteins is widely used by tumorigenic viruses, for example, E6 protein of HPV [118] or LT antigen of SV40 [119]. Reduced intracellular levels of HuR and/or AKA121 in 4Tluc2 cells expressing mutated RT_A variants would lead to overproduction of ROS beyond the levels controlled by Twist. Another explanation of overproduction of ROS in cells expressing mutant RT_As would be their aberrant capacity to induce Twist, which implies direct (not purely ROS mediated) involvement of HIV-1 RT in the regulation of Twist expression. The actual mechanism of the RT-induced production of ROS with possible involvement of HuR, AKAP 1, and/or other protein partners remains to be investigated.

In conclusion, the capacity to induce ROS appears to be a general property of HIV-1 reverse transcriptase; however, only the wild-type enzyme can efficiently induce activation of *Twist* to hamper oxidative stress. This property is responsible for the enhancement of tumorigenic and metastatic potential of RT-expressing cells. Our study casts light on one of the mechanisms of the direct potentiation of tumorigenesis by HIV resulting from the prooxidative activity of HIV-1 proteins. RT-mediated induction of ROS is of great interest also in the context of HIV-1 infection, as ROS are known to promote HIV-1 replication [120].

## Figures and Tables

**Table 1:** Kinetic parameters of the polymerase and RNase H activity of the variants of consensus RT of HIV-1 clade A FSU_A compared to RT of HIV-1 clade B HXB2 strain.

HIV-1 RT variants	Polymerase activity^∗^		RNase H activity^∗^	
	*K*_M_ (nM)	*V*_max_/mg RT (*μ*M/sec∗mg)	*K*_M_ (nM)	*V*_max_/mg RT (*μ*M/sec∗mg)
Wild-type RT of subtype B HXB2 strain (RT_B)	390 ± 95	4.0 ± 0.88	430 ± 60	21 ± 5
Consensus RT of subtype A (RT_A)	340 ± 100	7.5 ± 0.97 >RT_B (*p*=0.01)^∗∗^	470 ± 110	13 ± 4 <RT_B (*p* = 0.014)
Consensus RT of subtype A with primary mutations of resistance to nucleoside inhibitors K65R and M184 (RT_An)	320 ± 95	4.4 ± 0.18 <RT_A (*p* = 0.0055) <RT_Ann (*p* = 0.005)	400 ± 100	14 ± 2 <RT_B (*p* = 0.034)
Consensus RT of subtype A with primary mutations of resistance to nonnucleoside inhibitors K103N and G190S (RT_Ann)	400 ± 77	6.1 ± 0.5 >RT_B (*p* = 0.02) >RT_An (*p* = 0.005)	360 ± 60	8 ± 1 <RT_B (*p* = 0.0005) ≤RT_A (*p* = 0.09)

^∗^Results presented as mean ± SD in triplicate tests. ^∗∗^Significance of difference assessed by Fisher LSD test.

**Table 2:** Clones of 4T1luc2 expressing consensus RT of HIV-1 clade A FSU_A strain (RT_A) and its variants with primary mutations of resistance to NRTI (RT_An) and NNRTI (RT_Ann), obtained by lentiviral transduction of 4T1luc2 cells at different multiplicities of infection (MOI).

Enzyme	Polymerization *V*_max_^∗^, relative to RT_A^∗∗^	RNase H activity *V*_max_^∗^, relative to RT_A^∗∗^	Lentivirus	MOI	Derivative clone
RT-A	100%	100%	LV-RT-A	1	4T1luc2_RT-1.3
			LV-RT-A	5	4T1luc2_RT-5.3
			LV-RT-A	20	4T1luc2_RT-20.1

RT-An	59%	107%	LV-RT-An	1	4T1luc2_RT-An-1.4
			LV-RT-An	10	4T1luc2_RT-An-10.1

RT-Ann	81%	62%	LV-RT-Ann	1	4T1luc2_RT-Ann-1.5
			LV-RT-Ann	10	4T1luc2_RT-Ann-10.2

^∗^ *V*_max_ for polymerase and RNase H activities according to Table 1. ^∗∗^Mean *V*_max_ for the given RT_A variant divided by mean *V*_max_ for RT_A, in %.

## Data Availability

All data used to support the findings of this study are included within the article and the supplementary information file(s). Data not shown in the article used to support the findings of this study are available from the corresponding authors upon request.

## Supplementary Materials

Supplementary Materials Supplementary Table 1: table of primers. Primers used for confirmation of RT expression in 4T1luc2 derivatives and for relative quantification of epithelial to mesenchymal transition markers. Supplementary Table 2: table of semiquantitative PCR values. Relative expression levels of EMT markers in 4T1luc2 subclones expressing RT. Raw data used for correlation analysis and analysis of antioxidant treatment effect. Supplementary Table 3: table of statistical characteristics of multiple regression analysis of in vitro migration rate in wound healing assay. Statistical model of prediction of 4T1luc2 subclone migration in vitro in wound healing assay during 14 hours after the scratch was made based on different parameters, such as RT protein level, relative level of Twist expression, and migration in wound healing test during 14-18 hours after the scratch was made. Prediction was made by multiple regression analysis. Supplementary Figure 1: geographic and temporal distribution of RT amino acid sequences. Geographic origins and year of sample collection of the full-length sequences of reverse transcriptase (RT) of HIV-1 subtype A FSU_A strain used for the design of the amino acid consensus AFSUp66 (*n* = 44). Sequences were isolated from treatment-naïve patients free of known drug resistance mutations selected from HIV-1 sequence database and HIV Drug Resistance database. Supplementary Figure 2: schematic representation of lentiviral vector pRRLSIN.cPPT.PGK used for transduction of 4T1luc2 cell line with the polynucleotide sequences encoding the consensus RT_A with or without drug resistance mutations. Lentiviral transduction resulted in seven 4T1luc2 derivative clones expressing different levels of three RT variants. Supplementary Figure 3: RNase H activity of consensus RT_A variants with and without drug resistance mutations. Graphs demonstrate RNase activity of RT_A variants, firstly as products of RNase H cleavage of the substrate after analysis by PAGE and secondly concentration dependence of RNase H activity of the variants of consensus RT of clade A FSU_A strain with and without drug resistance mutations. The respective kinetic parameters are described in the main text. Supplementary Figure 4: graphs comparing the levels of ROS in 4T1luc2 cells expressing RT_A, parental 4T1luc2 cells, and immortal NIH3T3 cell line. Increased level of produced ROS by 4T1luc2 derivative clones expressing RT compared to the parental cells, measured as an increase of relative intensity of DCFH2-DA/DAPI fluorescent signal. Cells treated with H_2_O_2_ or *tert*-butylhydroquinone (tBHQ) were used as positive control, and cells treated with antioxidants tocopherol and N-acetyl-L-cysteine (NAC) as negative control. Increased level of ROS production in the parental 4T1luc2 resulted in increased basal level of lipid peroxidation, measured by MDA level in cell lysates, as compared to the immortal NIH3T3 cell line known to produce low levels of ROS. Supplementary Figure 5: wound healing assay. Panels represent light microscopy images of the gap area at 0, 14, and 18 hours after making the scratch. Timeline was settled to measure the migration rate before and after doubling time (approx. 14 hours). Original .tif files of high resolution are available at the following link https://drive.google.com/drive/folders/1a4VbLzFYeny9LDf5iTxyhSmSanIesLl1?usp=sharing. Supplementary Figure 6: graphs showing that expression of Twist on day 14, but not on day 3 of cell culture, correlates to the level of expression of RT. Treatment of cells with antioxidant N-acetyl-L-cysteine (NAC) leads to the loss of correlation between expression of RT and Twist on day 14, demonstrating that induction of expression of Twist depends on the production of ROS. Graphs are supplemented with *r*, *r*^2^, and *p* values of the Spearman correlation test. Supplementary Figure 7: graphs showing correlation between the levels of expression of Twist and other EMT markers. On day 14 of cell culture, level of expression of Twist correlated with the levels of expression of EMT markers N-cadherin, Vimentin, and Snail, but not with the expression of E-cadherin. Textbox in the figure shows correlation values (*r*, *r*^2^, and *p*) measured by the Spearman correlation test. Supplementary Figure 8: graphs showing correlation between the levels of ROS and the levels of expression of EMT markers N-cadherin on day 3 and Vimentin on day 14 of cell culture. ROS levels are presented either per cell or relative to the levels exhibited by the parental 4T1luc2 cells. Textbox in the figure shows correlation values (*r*, *r*^2^, and *p*) measured by the Spearman correlation test.
