# Supplementary material for: HIV-1 Reverse Transcriptase Promotes Tumor Growth and Metastasis Formation via ROS-Dependent Upregulation of Twist
Source: Oxid Med Cell Longev. 2019 Dec 2;2019:6016278. doi: 10.1155/2019/6016278 (PMC6915010; doi:10.1155/2019/6016278)
Supplement: Supplementary Materials — Supplementary Table 1: table of primers. Primers used for confirmation of RT expression in 4T1luc2 derivatives and for relative quantification of epithelial to mesenchymal transition markers. Supplementary Table 2: table of semiquantitative PCR values. Relative expression levels of EMT markers in 4T1luc2 subclones expressing RT. Raw data used for correlation analysis and analysis of antioxidant treatment effect. Supplementary Table 3: table of statistical characteristics of multiple regression analysis of in vitro migration rate in wound healing assay. Statistical model of prediction of 4T1luc2 subclone migration in vitro in wound healing assay during 14 hours after the scratch was made based on different parameters, such as RT protein level, relative level of Twist expression, and migration in wound healing test during 14-18 hours after the scratch was made. Prediction was made by multiple regression analysis. Supplementary Figure 1: geographic and temporal distribution of RT amino acid sequences. Geographic origins and year of sample collection of the full-length sequences of reverse transcriptase (RT) of HIV-1 subtype A FSU_A strain used for the design of the amino acid consensus AFSUp66 (n = 44). Sequences were isolated from treatment-naïve patients free of known drug resistance mutations selected from HIV-1 sequence database and HIV Drug Resistance database. Supplementary Figure 2: schematic representation of lentiviral vector pRRLSIN.cPPT.PGK used for transduction of 4T1luc2 cell line with the polynucleotide sequences encoding the consensus RT_A with or without drug resistance mutations. Lentiviral transduction resulted in seven 4T1luc2 derivative clones expressing different levels of three RT variants. Supplementary Figure 3: RNase H activity of consensus RT_A variants with and without drug resistance mutations. Graphs demonstrate RNase activity of RT_A variants, firstly as products of RNase H cleavage of the substrate after analysis by PAGE and secondly conc [file 6016278.f1.pdf]

HIV-1 reverse transcriptase promotes tumor growth and metastasis formation via ROS-dependent upregulation of Twist

Ekaterina Bayurova[1,2*], Juris Jansons[3,4], Dace Skrastina[3,4], Olga Smirnova[5], Dzeina Mezale[3], Anastasia Kostyusheva[6], Dmitry Kostyushev[6], Stefan Petkov[7], Philip Podschwadt[7], Vladimir Valuev-Elliston[5], Sviataslau Sasinovich[7], Sergey Korolev[8], Per Warholm[9], Anastasia Latanova[1,5], Elizaveta Starodubova[1,5], Amir Tukhvatulin[1], Oleg Latyshev[1], Renat Selimov[10], Pavel Metalnikov[10], Alexander Komarov[10], Olga Ivanova[5], Tatiana Gorodnicheva[11], Sergey Kochetkov[5], Marina Gottikh[8], Ilze Strumfa[3], Alexander Ivanov[5], Ilya Gordeychuk[1,2,12], and Maria Isaguliants[1,2,3,7]*

[1]*NF Gamaleya Research Center of Epidemiology and Microbiology, Moscow, Russia; ekaterinapankova48135@gmail.com (EB); oleglat80@mail.ru (OL); lab.gord@gmail.com (IG); aalatanova@gmail.com (AL); maria.issagouliantis@rsu.lv (MI);*

[2]*Chumakov Federal Scientific Center for Research and Development of Immune-and-Biological Products of Russian Academy of Sciences, Moscow, Russia; ekaterinapankova48135@gmail.com (EB); lab.gord@gmail.com (IG); maria.issagouliantis@rsu.lv (MI);*

[3]*Department of Pathology, Riga Stradins University, Riga, Latvia; daceskr@biomed.lu.lv (DS); juris.jansons@rsu.lv (JJ); dzeina.mezale@rsu.lv (DM); ilze.strumfa@rsu.lv (IS); maria.issagouliantis@rsu.lv (MI);*

[4]*Latvian Biomedical Research and Study Centre, Riga, Latvia; jansons@biomed.lu.lv (JJ); daceskr@biomed.lu.lv (DS);*

[5]*Engelhardt Institute of Molecular Biology, Russian Academy of Sciences, Moscow, Russia; o.smirnova.imb@gmail.com (OS); estarodubova@yandex.ru (ES); Olgaum@yandex.ru (OI);*

gansfaust@mail.ru (VVE); kochet@eimb.ru (SNK); aivanov@yandex.ru (AI);

[6]*National Medical Research Center for Tuberculosis and Infectious Diseases, Moscow, Russia, e-mails: kostyusheva_ap@mail.ru (AK); dkostushev@gmail.com (DK)*

[7]*Department of Microbiology, Tumor and Cell Biology, Karolinska Institutet, Stockholm, Sweden; stefan.petkov@ki.se (SP); philip.podschwadt@uk-essen.de (PP); sviataslau.s@gmail.com (SS); maria.issagouliantis@ki.se (MI);*

[8]*Chemistry Department and Belozersky Institute of Physico-chemical Biology, Lomonosov Moscow State University, Moscow, Russia; spkorolev@mail.ru (SK); gottikh@belozersky.msu.ru (MG);*

[9]*Science for Life Laboratory, Stockholm University, Stockholm, Sweden; e-mail: per.warholm@gmail.com (PW);*

[10]*Russian State Center for Quality and Standardization of veterinary drugs and feed (VGNKI), Moscow, Russia; e-mails: selimov@vgnki.ru (RS), metalnikov@vgnki.ru (PM), komarov1965@hotmail.com (AK)*

[11]*Evrogen, Moscow, Russia; e-mail: tatiana.gorod@evrogen.ru (TG).*

[12]*Sechenov First Moscow State Medical University, Moscow, Russia; lab.gord@gmail.com (IG).*

* Corresponding authors, Ekaterina Bayurova (*ekaterinapankova48135@gmail.com)* and Maria Isaguliants (*maria.issagouliantis@rsu.lv*).

SUPPLEMENTARY MATERIALS

Supplementary Table 1 – Table of primers. Primers used for confirmation of RT expression in 4T1luc2 derivatives and for relative quantification of epithelial to mesenchymal transition markers.

Supplementary Table 2 – **Table of semi-quantitative PCR values**. Relative expression levels of EMT markers in 4T1luc2 subclones expressing RT. Raw data used for correlation analysis and analysis of antioxidant treatment effect.

Supplementary Table 3. – Table of statistical characteristics of multiple regression analysis of *in vitro* migration rate in wound healing assay. Statistical model of prediction of 4T1luc2 subclones migration *in vitro* in wound healing assay during 14 hours after the scratch made based on different parameters, such as RT protein level, relative level of Twist expression and migration in wound healing test during 14-18 hours after the scratch was made. Prediction was made by multiple regression analysis.

Supplementary Figure 1 – Geographic and temporal distribution of RT amino acid sequences. Geographic origins and year of sample collection of the full-length sequences of reverse transcriptase (RT) of HIV-1 subtype A FSU_A strain used for the design of the amino acid

consensus AFSUp66 (n=44). Sequences were isolated from treatment naïve patients free of known drug resistance mutations selected from HIV-1 sequence database and HIV Drug Resistance database.

Supplementary Figure 2 – Schematic representation of lentiviral vector pRRLSIN.cPPT.PGK used for transduction of 4T1luc2 cell line with the polynucleotide sequences encoding the consensus RT_A with or without drug resistance mutations. Lentiviral transduction resulted in seven 4T1luc2 derivative clones expressing different levels of three RT variants.

Supplementary Figure 3 – RNase H activity of consensus RT_A variants with and without drug resistance mutations. Graphs demonstrate RNase activity of RT_A variants, firstly as products of RNAse H cleavage of the substrate after analysis by PAGE, and secondly, concentration dependence of RNase H activity of the variants of consensus RT of clade A FSU_A strain with and without drug resistance mutations. The respective kinetic parameters are described in the main text.

Supplementary Figure 4 – Graphs, comparing the levels of ROS in 4T1luc2 cells expressing RT_A, parental 4T1luc2 cells and immortal NIH3T3 cell line. Increased level of produced ROS by 4T1luc2 derivative clones expressing RT compared to the parental cells, measured as an increase of relative intensity of DCFH2-DA/DAPI fluorescent signal. Cells treated with H_2O_2 or tert-butylhydroquinone (tBHQ) were used as positive control, and cells treated with antioxidants tocopherol and N-acetyl-L-cysteine (NAC), as negative control. Increased level of ROS production in the parental 4T1luc2, resulted in increased basal level of lipid peroxidation, measured by MDA level in cell lysates, as compared to the immortal NIH3T3 cell line known to produce low levels of ROS.

Supplementary Figure 5 – Wound healing assay. Panels represent light microscopy images of the gap area at 0, 14 and 18 hours after making the scratch. Time line was settled to measure the migration rate before and after doubling time (approx. 14 hours). Original .tif files of high resolution are available at the following link https://drive.google.com/drive/folders/1a4VbLzFYeny9LDf5iTxyhSmSanIesLl1?usp=sharing.

Supplementary Figure 6 – Graphs showing that expression of *Twist* on day 14, but not on day 3 of cell culture, correlates to the level of expression of RT. Treatment of cells with antioxidant N-acetyl-L-cysteine (NAC) lead to loss of correlation between expression of RT and Twist on day 14, demonstrating that induction of expression of Twist depends on the production of ROS. Graphs are supplemented with r, r^2 and p values of the Spearman correlation test.

Supplementary Figure 7 – Graphs showing correlation between the levels of expression of *Twist* and other EMT markers. On day 14 of cell culture, level of expression of *Twist* correlated with the levels of expression of EMT markers *N-cadherin*, *Vimentin* and *Snail*, but not with the expression of *E-cadherin*. Textbox in the figure shows correlation values (r, r^2 and p) measured by the Spearman correlation test.

Supplementary Figure 8 – Graphs showing correlation between the levels of ROS and the levels of expression of EMT markers *N-cadherin* on day 3 and *Vimentin* on day 14 of cell culture. ROS levels are presented either per cell, or relative to the levels exhibited by the parental 4T1luc2 cells. Textbox in the figure shows correlation values (r, r^2 and p) measured by the Spearman correlation test.

Supplementary Table 1. List of primers used in the experiments. Fw – forward, rev – reverse.

Target sequence (gene)	Direction	Primer sequence
HPRT1	fw	5'-GGCCAGACTTTGTTGGATTT-3'
	rev	5'-CAGATTCAACTTGCGCTCAT-3'
Vimentin	fw	5'-CGGAAAGTGGAATCCTTGCA-3'
	rev	5'-CACATCGATCTGGACATG CTG T-3'
N-cadherin	fw	5'-AGGGTGGACGTCATTGTA GC-3'
	rev	5'-CTGTTGGGGTCTGTC-3'
Snail	fw	5'-AACCCACTCGGATGTGAAGAG-3'
	rev	5'-GACTCTTGGTGCTTGTGGAG-3'
Twist	fw	5'-CAGCGGGTCATGGCTAACG-3'
	rev	5'-TTGCTCAGCTTGTCCGAGG-3'
E-cadherin	fw	5'-ACGTATCAGGGTCAAGTGCC-3
	rev	5'-ATCATTGGTCGTGGGGTCTG-3'
Reverse transcriptase of HIV-1 FSU_A strain (RT_A)	fw	5'-CTGGAGCTTGCTGAGAATAGAG-3'
	rev	5'-CACTGGTCCTGTCCTTGTTT-3'
Lentiviral insertion (PGKseq)*	fw	5`- GGTGTTCCGCATTCTGCAAG – 3`
Lentiviral insertion (LVT-200R)*	rew	5` -GACAACGGGCCACAACTCC – 3`

* - positions of primers used to confirm the insertion of lentiviruses encoding RT_A variants are depicted in Supplementary Figure 2.

Supplementary Table 2. Expression of epithelial and mesenchymal markers at days 3 and 14 of cell culture. Data shows fold increase of expression of *E cadherin, Vimentin, N cadherin, Twist* and *Snail* genes as compared to the level of expression of these genes by the parental 4T1luc2 cell line on days 3 and 14 of cell culture. Each value represents the mean of three independent measurements±SD

	E cad		N cad		vim		twist		snail	
Day	3	14	3	14	3	14	3	14	3	14
4T1luc2	1	1	1	1	1	1	1	1	1	1
4T1luc2_RT-1.3	1,29±0,03	0,99±0,28	2,32±0,13	3,15±1,05	0,29±0,03	3,47±1,13	3,35±0,06	1,78±0,64	1,70±0,32	2,71±0,81
4T1luc2_RT-5.3	0,83±0,01	1,32±0,03	1,29±0,22	0,95±0,17	1,02±0,08	0,95±0,23	0,97±0,08	2,49±0,62	1,21±0,05	1,04±0,16
4T1luc2_RT-20.1	0,83±0,08	1,89±0,16	1,60±0,08	6,87±1,37	0,65±0,03	8,96±1,15	0,80±0,07	3,65±0,51	1,26±0,09	5,15±0,7
4T1luc2_RT-An-1.4	0,52±0,06	0,80±0,09	2,48±0,14	0,67±0,05	0,52±0,01	2,83±0,41	3,21±0,11	1,30±0,17	1,45±0,03	1,03±0,11
4T1luc2_RT-An-10.2	0,59±0,07	0,30±0,06	0,65±0,02	0,45±0,08	0,27±0,02	1,94±0,06	0,51±0,02	0,24±0,04	0,54±0,02	0,37±0,05
4T1luc2_RT-Ann-1.5	1,50±0,19	3,14±0,09	0,46±0,08	1,13±0,49	1,48±0,27	0,78±0,16	5,38±0,7	1,13±0,23	1,50±0,3	1,89±0,71
4T1luc2_RT-Ann-10.2	1,38±0,38	1,95±0,12	2,29±0,36	3,23±1,09	0,22±0,03	7,93±1,86	0,66±0,06	1,62±0,43	1,67±0,3	3,09±1,03

Supplementary Table 3. Prediction of the rate of migration of 4T1luc2 cells expressing RT_A variants in the first 14 hours of wound healing assay using multiple regression analysis.

Statistical parameters	Model A*	Model B**	Model C***	Model D****
Multiple R	0,962466214	0,944540631	0,928125251	0,961466674
Multiple R^2	0,926341214	0,892157003	0,861416481	0,924418166
Adjusted R^2	0,828129499	0,811274755	0,805983074	0,86773179
F(4,3)	9,43208471	11,0303191	15,5396632	16,3075898
p	0,047768984	0,0210059918	0,00714955386	0,0104373489
Std.Err. of Estimate	0,363574551	0,380984936	0,386289243	0,318948485

Parameters of the models: * — relative ROS; distance covered by the cells in the interval between 14 and 18 hours after the scratch (%); relative levels of Twist mRNA on day 14; RT expression (pg/cell); ** — relative ROS; distance covered by the cells in the interval between 14 and 18 hours after the scratch (%); relative levels of Twist mRNA on day 14; *** — relative ROS; distance covered by the cells in the interval between 14 and 18 hours after the scratch (%); **** — relative ROS; relative levels of Twist mRNA on day 14; RT expression (pg/cell). Statistically significant values are highlighted in red.

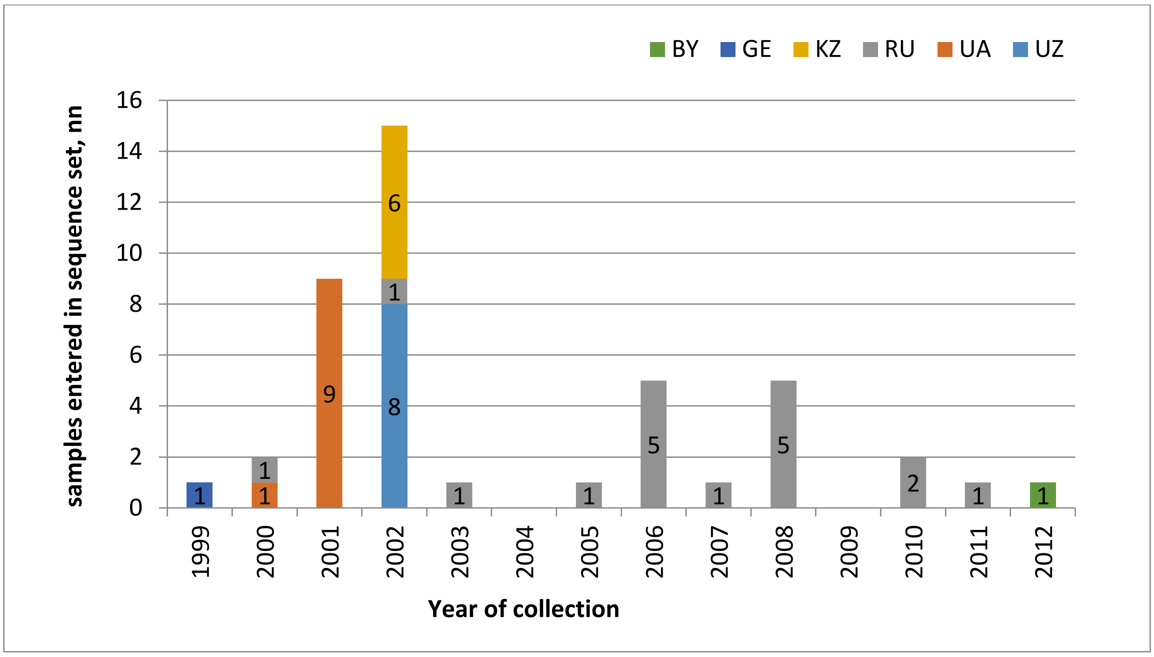

Supplementary Figure 1. Geographic origins and year of sample collection of the full-length sequences of reverse transcriptase of HIV-1 subtype A FSU_A strain (RT_A) used in the design of the amino acid consensus AFSUp66 (n=44). Figures within the bars represent the numbers of RT sequences originating from a given country in a given year.

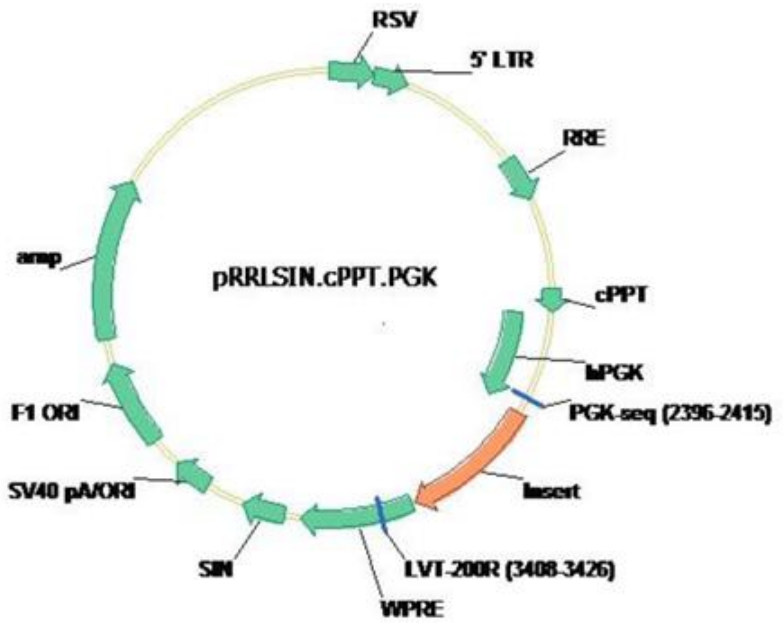

Supplementary Figure 2. Lentiviral vector pRRLSIN.cPPT.PGK used for transduction of 4T1luc2 cell line. Position of insertion of RT-A, RT-An or RT-Ann genes marked in red. PGK-seq and LVT-200R (Supplementary Table 1) PCR primers were used to confirm the insertion of lentiviruses encoding RT_A variants into genome of 4T1luc2 cells.

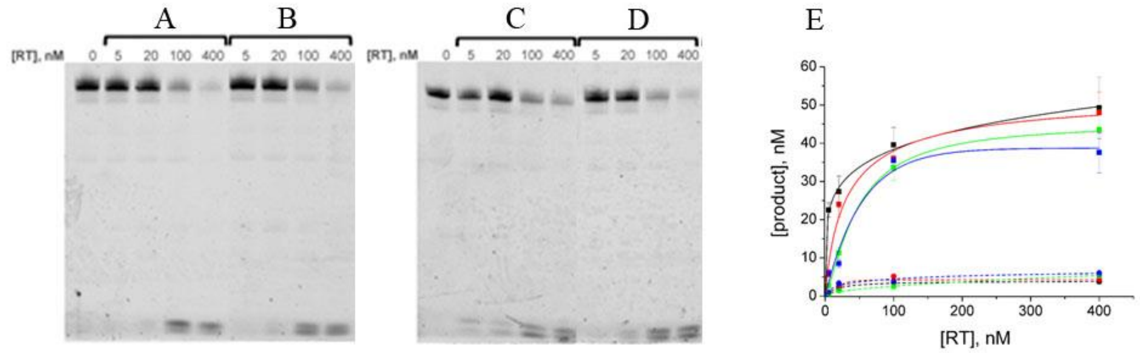

Supplementary Figure 3. Concentration dependence of RNase H activity of the variants of consensus RT of clade A FSU_A strain with and without drug resistance mutations. Analysis of RNA degradation by RNase H activity in 20% PAAG-7M Urea. HIV HXB2 (A), consensus RT_A (B), RT_A with mutations of resistance to NRTI (C) and NNRTI (D). (E) Quantification of the results: RT of HIV-1 HXB2 (black), consensus RT_A (red); consensus RT_A with NRTI resistance mutations M184V and K65R (RT_An; green); consensus RT_A with NNRTI resistance mutations K103N and G190S (RT_Ann; blue). Solid lines with square dots designate active, and dashed lines with circles, RT variants with mutations of inactivation, demonstrating that inactivation leads to complete loss of the activity of RNAse H. Quantification represents the results of three independent runs.

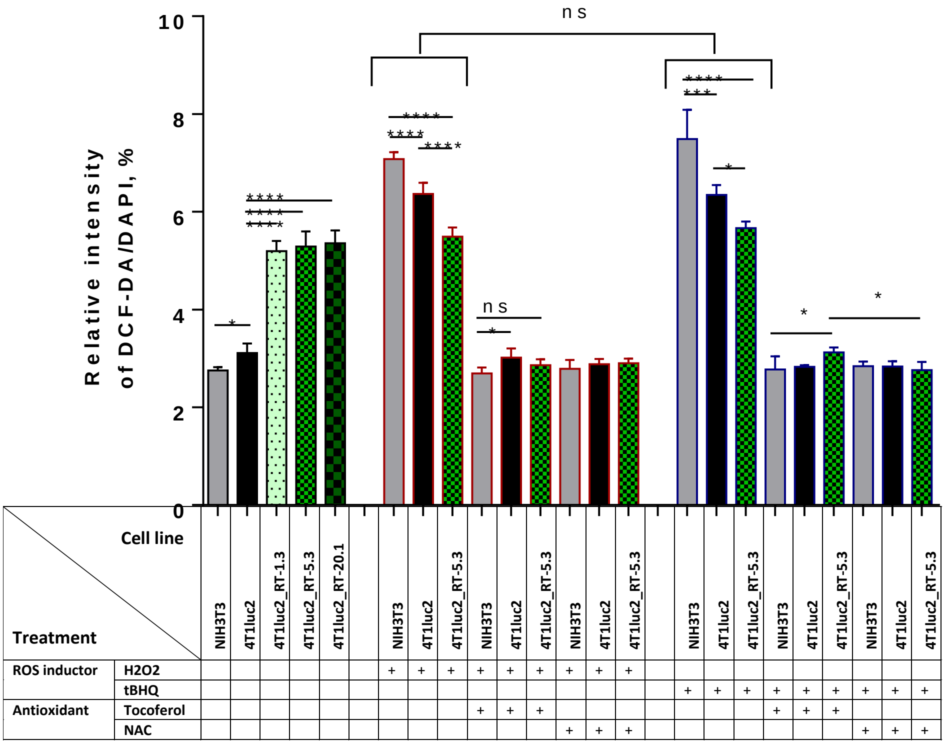

(A)

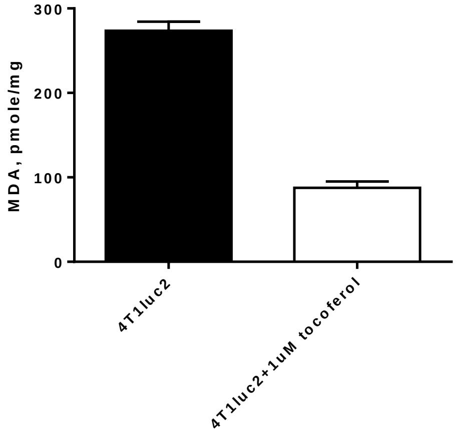

(B)

Supplementary Figure 4. Levels of ROS produced in 4T1luc2 cells expressing RT_A variants in the presence and absence of antioxidants tocoferol and N-acetyl-L-cysteine (NAC) and ROS inducers H_2O_2 and tert-butylhydroquinone (tBHQ). Parental 4T1luc2 and control NIH3T3 cells are given for comparison. (A) 4T1luc2 cells expressing RT_A variants induce ROS. Levels of ROS are significantly higher than those registered for the control immortal cell line NIH3T3 or parental 4T1luc2 cells. Levels of ROS production by RT_A expressing 4T1luc2 cells cannot be further increased even by treatment of cells with ROS inducers H_2O_2 and tBHQ. In all the cases, treatment with antioxidants tocoferol or NAC quenches the production of ROS to the levels characteristic to the parental 4T1luc2 cells. (B) Effect of antioxidants on lipid

peroxidation, measured by MDA level in cell lysates. All values are expressed as mean±SD. Analyzed by one-way ANOVA followed by unpaired t-test, or by Kruskal-Wallis test followed by Mann-Whitney test * - $p<0,05$, ** - $p<0,005$, *** - $p<0,0005$, **** - $p<0001$.

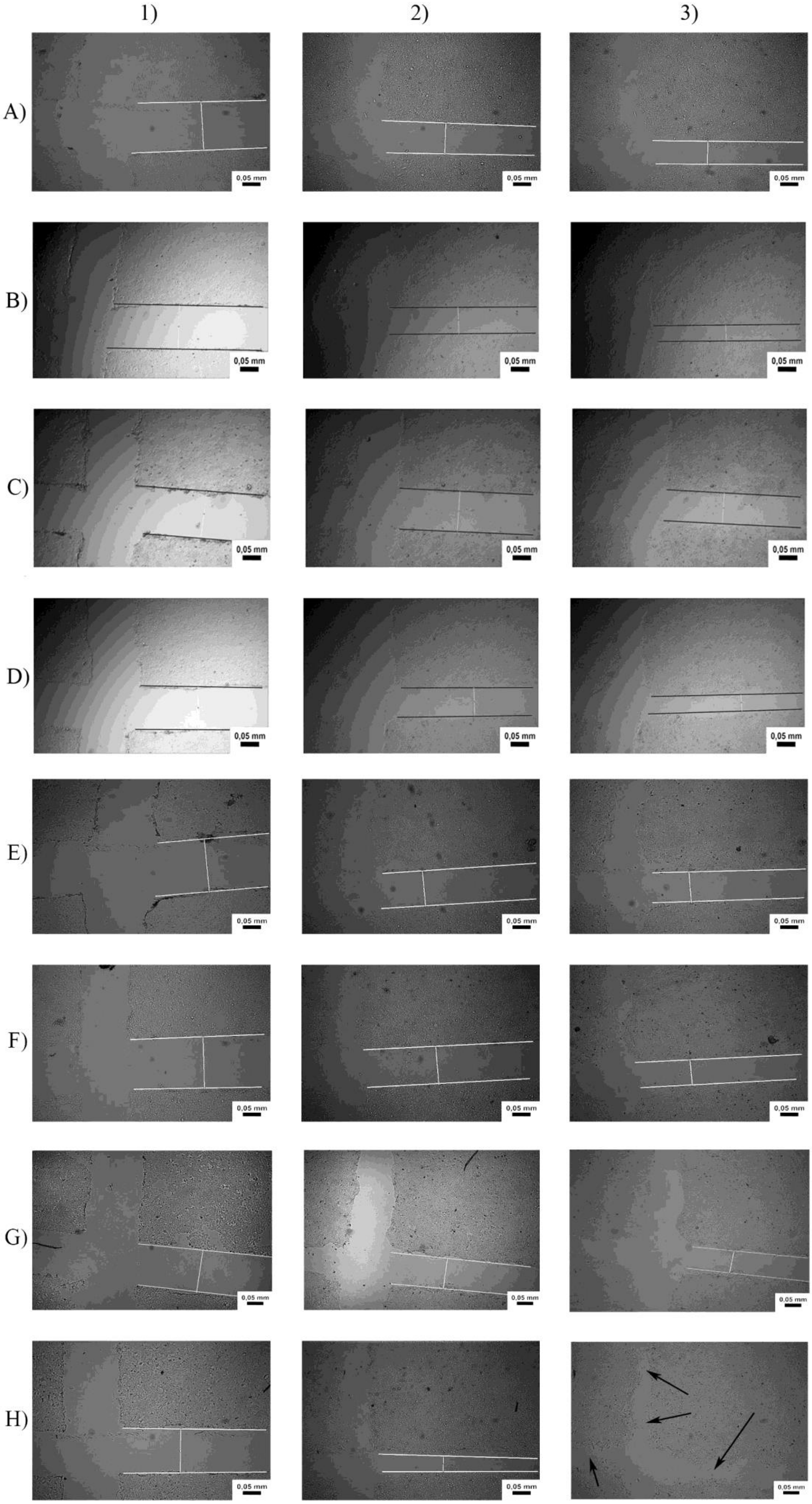
1)
2)
3)
A)
B)
C)
D)
E)
F)
G)
H)
0,05 mm

I) 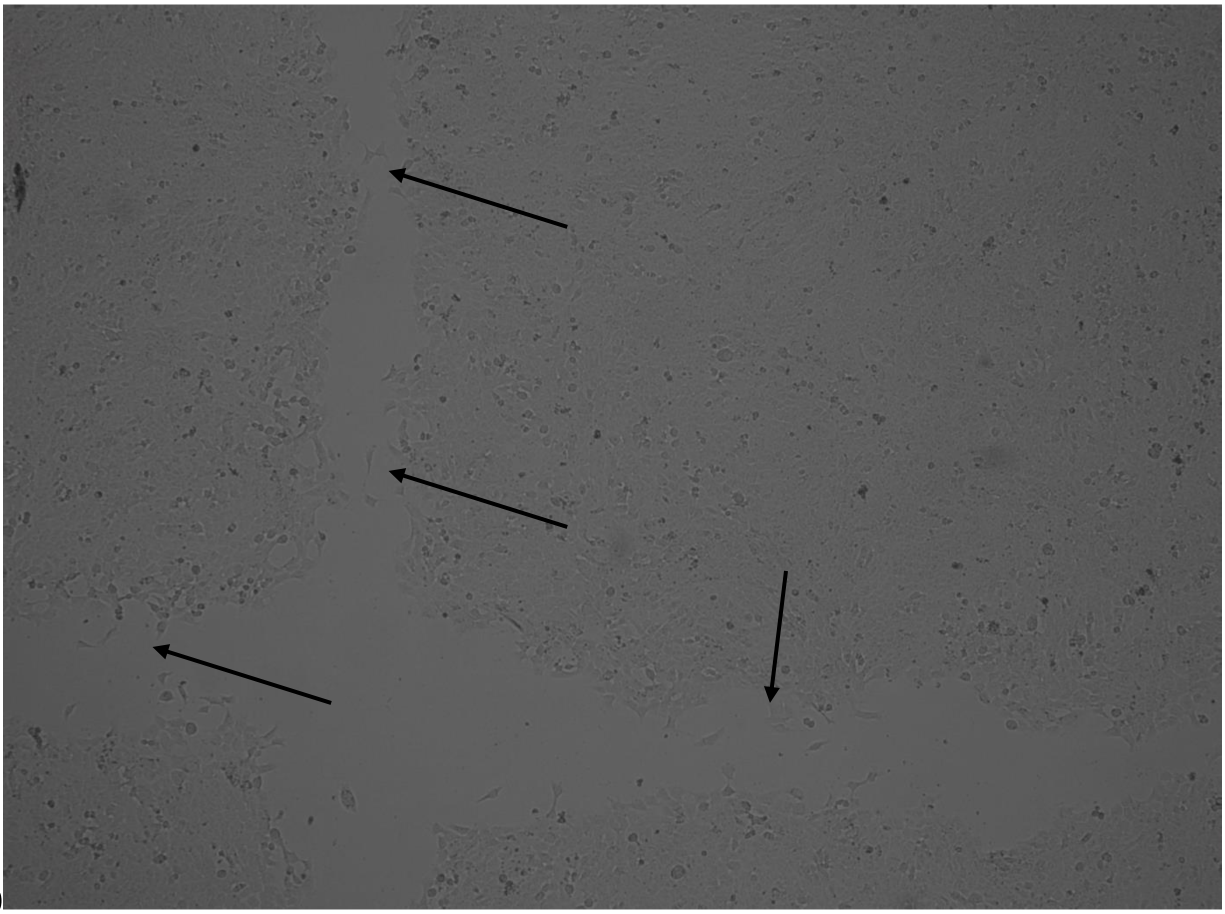

Supplementary Figure 5. Wound healing assay. Registration by light microscopy of the gap size formed by the parental 4T1luc2 (A) and derivative clones 4T1luc2_RT-1.3 (B), 4T1luc2_RT-5.3 (C), 4T1luc2_RT-20.1 (D), 4T1luc2_RT-An-1.4 (E), 4T1luc2_RT-An-10.1 (F), 4T1luc2_RT-Ann-1.5 (G), 4T1luc2_RT-Ann-10.2 (H). Panels 1, 2 and 3 represent 0, 14 and 18 hours after the scratch, respectively. The edges of the gap are marked by lines. (I) Enlarged panel H3 illustrates 4T1luc2_RT-Ann-10.2 18 hours after the scratch. Single cell migration phenotype is indicated by arrows.

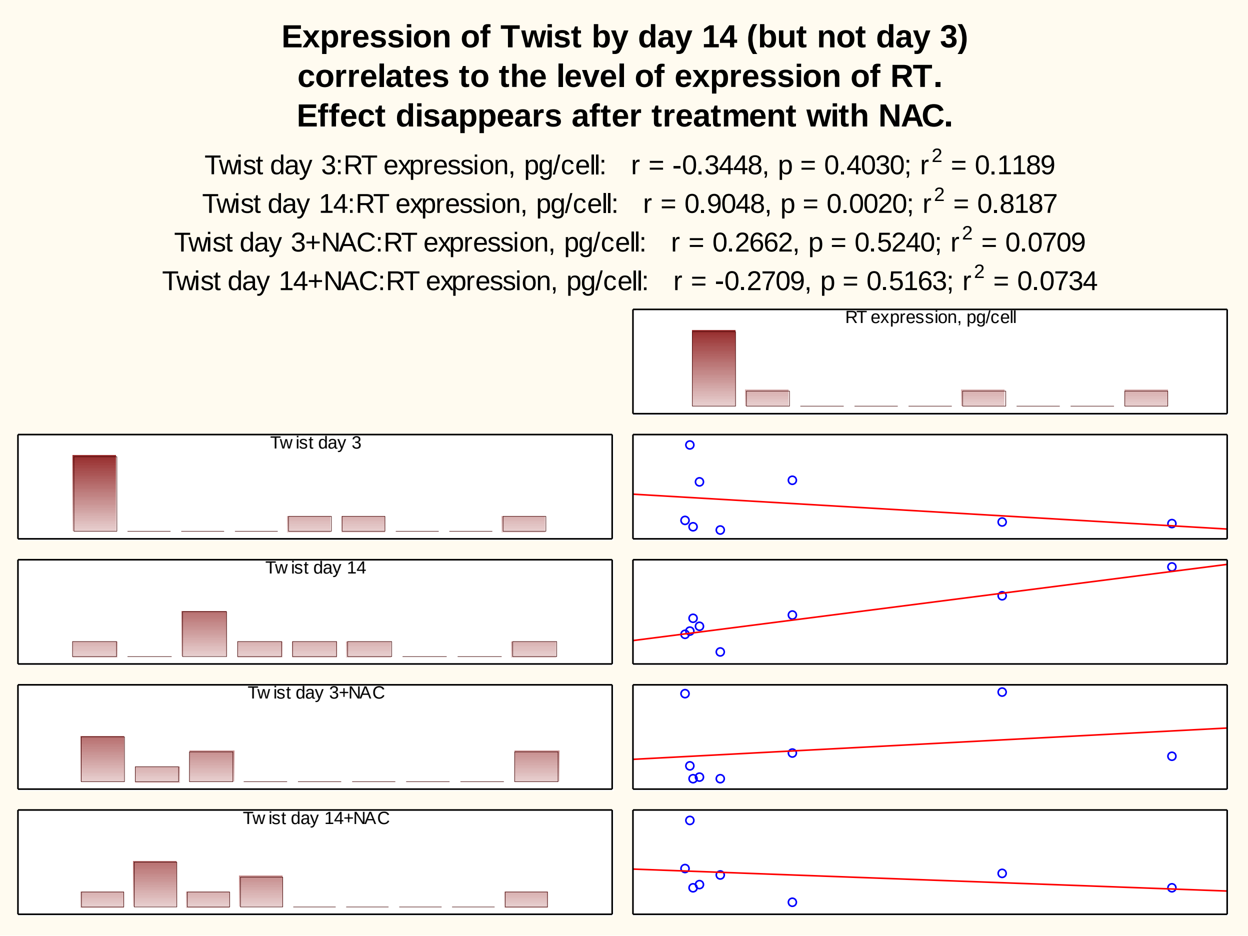

Supplementary Figure 6. Expression of *Twist* on day 14, but not on day 3 of cell culture, correlates to the level of expression of RT. Effect disappeared after treatment of cells with antioxidant NAC, demonstrating that induction of expression of Twist depends on the production of ROS. Textbox in the figure shows correlation values (r, r^2 and p) measured by the Spearman correlation test (STATISTICA AXA 10). Expression level of *Twist* was normalized to the expression of the reference gene *HPRT1* and accounted as fold increase compared to the levels exhibited by the parental 4T1luc2 NAC treatment was done as in Supplementary Figure 4.

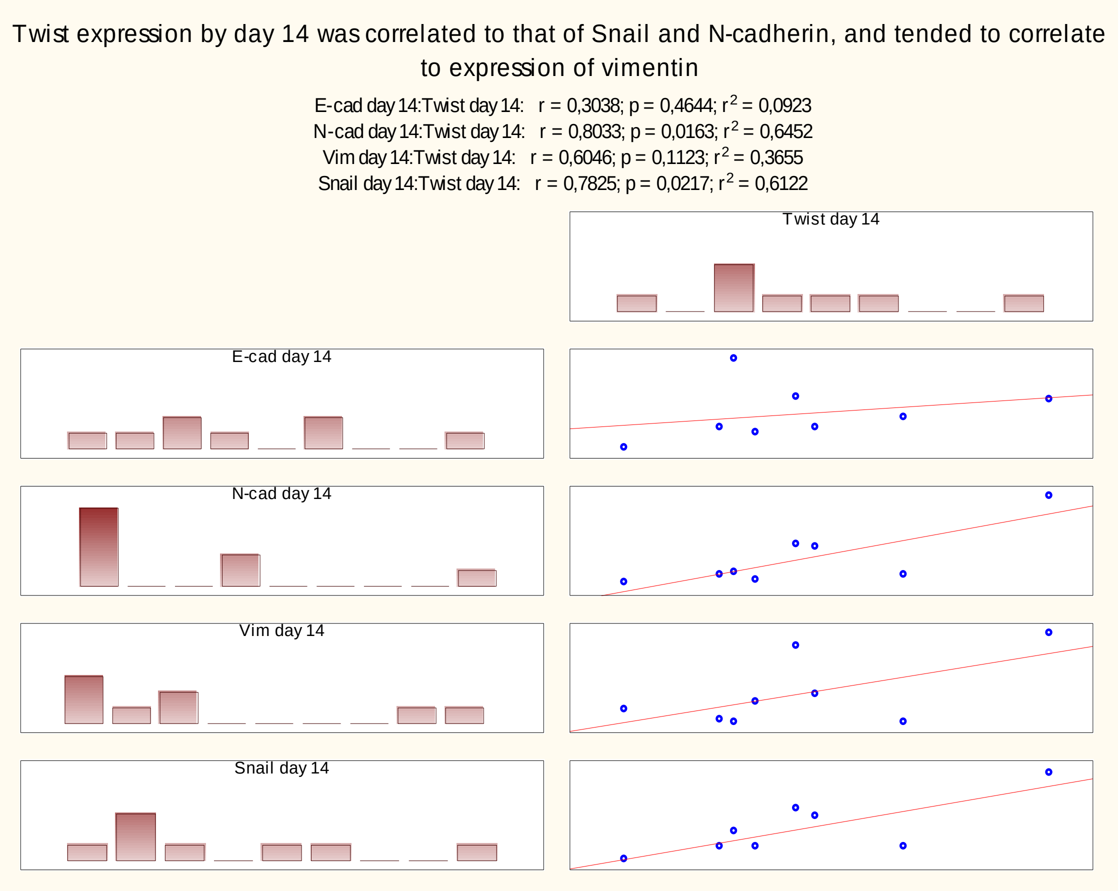

Supplementary Figure 7. Correlation between the levels of expressions of *Twist* and other EMT markers. On day 14 of cell culture, level of expression of *Twist* correlated with the levels of expression of EMT markers *N-cadherin*, *Vimentin* and *Snail*, but not with the expression of *E-cadherin*. Textbox in the figure shows correlation values (r, r^2 and p) measured by the Spearman correlation test (STATISTICA AXA 10). Expression levels of *Twist*, *E-cadherin*, *N-cadherin*, *Vimentin* and *Snail* were normalized against the expression of *HPRT1* and calculated as fold increase compared to the levels exhibited by the parental 4T1luc2 cells.

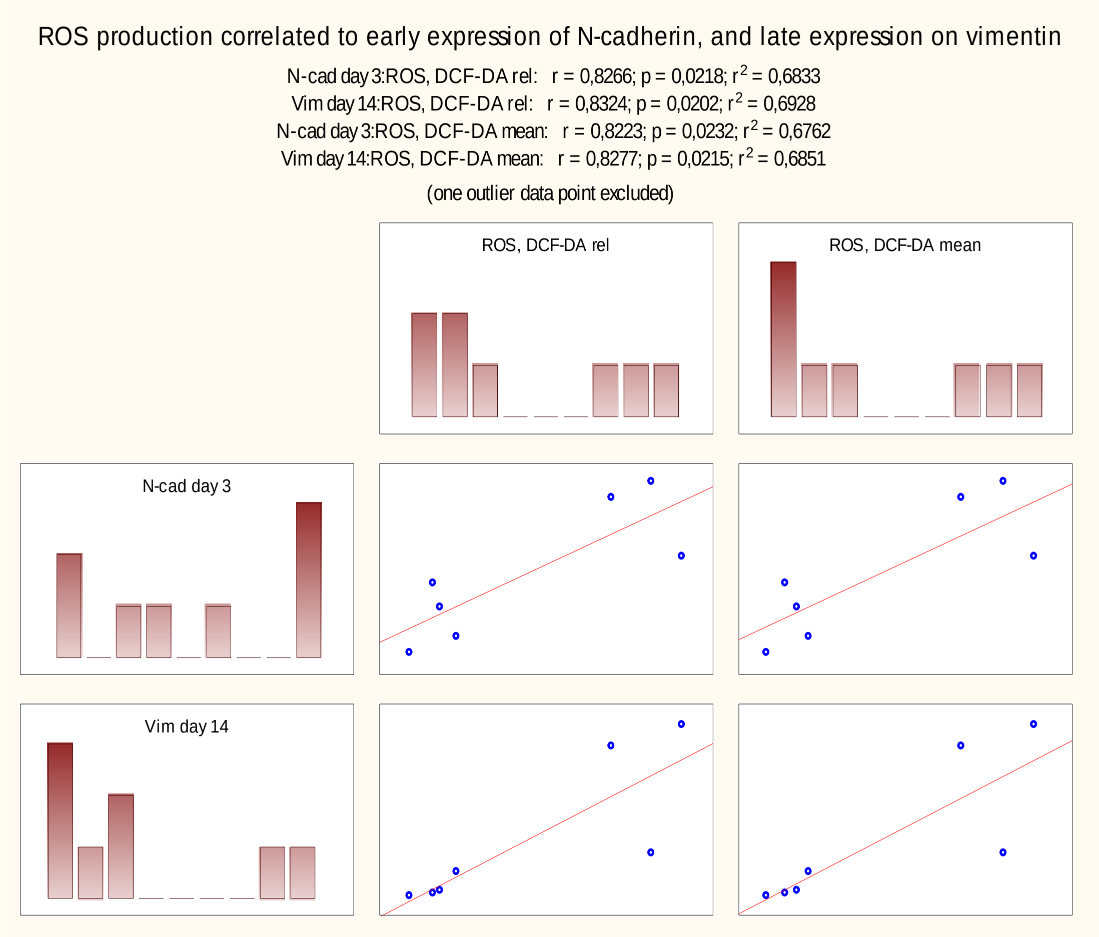

Supplementary Figure 8. Correlation of ROS production and levels of expression of EMT markers *N-cadherin* and *Vimentin*. Correlation between the level of ROS production per cell (ROS, DCF-DA mean) or relative to parental 4T1luc2 line (ROS, DCF-DA rel) and expression levels of *N-cadherin* on day 3 and *Vimentin* on day 14 of cell culture. Expression levels of *N-cadherin* and *Vimentin* were normalized against the expression of *HPRT1* and calculated as fold increase compared to the levels exhibited by the parental 4T1luc2 cells. Textbox in the figure shows correlation values (r, r^2 and p) measured by the Spearman correlation test (STATISTICA AXA 10).
